# Molecular Dynamics Study on Tetraglyme Solutions of Two Lithium Salts with Isomeric Anions: LiTFSI and LiFPFSI

**DOI:** 10.1021/acs.jpcb.5c07631

**Published:** 2026-01-30

**Authors:** Piotr Kubisiak, Chiara Nicotri, Andrzej Eilmes

**Affiliations:** † Faculty of Chemistry, 37799 Jagiellonian University, Gronostajowa 2, 30-387 Kraków, Poland; ‡ Department of Applied Science and Technology, 19032 Politecnico di Torino, Corso Duca degli Abruzzi 24, 10129 Torino, Italy

## Abstract

We used classical molecular dynamics simulations with a polarizable force field to compare Li-ion-conducting electrolytes based on tetraglyme as a solvent and two isomeric salts, LiTFSI and LiFPFSI. Analysis of the structural information and dynamics of the systems reveals that, for both salts, very stable [Li­(tetraglyme)]^+^ solvates form in the electrolyte. For an equimolar salt/solvent composition, solutions exhibit properties of a solvate ionic liquid. In LiFPFSI electrolytes, the Li-anion interactions are slightly weaker than those in LiTFSI solutions, resulting in more stable Li^+^ solvates. Although the diffusion coefficients of ions are similar for both salts, the ionic conductivities of LiFPFSI electrolytes estimated from the simulations are 40–70% larger than the conductivities of LiTFSI solutions. This enhancement originates from constructive contributions to the conductivity arising from anticorrelated motions of cations and anions, a feature characteristic of ionic liquids. Therefore, the detailed analysis of ion–ion correlations is necessary for a deeper understanding of ion transport in concentrated solutions.

## Graphical abstract

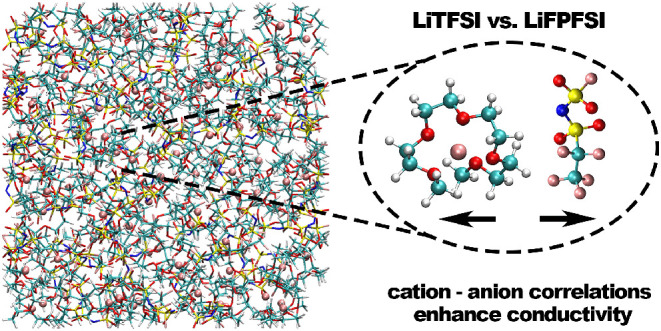

## 1 Introduction

The sustainable development of modern society requires efficient energy storage devices to address the intermittency of power production from renewable resources and to meet the growing demand for portable energy storage devices, finding numerous applications in everyday life, from smartphones to electric vehicles. Although lithium-ion batteries (LIBs),^1–3^ available since the 1990s, have achieved great commercial success, the search continues for new devices that are safer, more environmentally friendly, and more efficient, stimulating ongoing research in energy storage technology.

A key component of a metal-ion battery is the ion-conducting electrolyte—a salt solution in a molecular solvent, an ionic liquid (IL), or a polymer matrix.^4–6^ Commercial LIBs typically use lithium hexafluorophosphate in organic carbonates (ethylene carbonate and propylene carbonate).^4^ Nevertheless, several other salts are experimentally investigated as potential promising alternatives, including those with weakly coordinating anions such as lithium bis­(trifluoromethanesulfonyl)­imide (LiTFSI).^7^ LiTFSI is often used in ethereal solutions with oligoglymes as the solvents and in polymer electrolytes based on poly­(ethylene oxide) (PEO). Of special interest are the properties of LiTFSI solutions in triglyme or tetraglyme (G4), in which stable [Li­(glyme)]^+^ complexes are formed, and thus at the equimolar salt/solvent ratio, these systems exhibit solvate ionic liquid (SIL) behavior.^8–12^

Numerous computational studies have investigated conformations of TFSI anions,^13–17^ their interactions with lithium cations^10,18–20^ and vibrational spectra used experimentally to monitor the coordination of anions in the electrolyte.^8,13,14,16,18,19^ Both classical and first-principles molecular dynamics (MD) simulations have been employed to analyze Li^+^ binding as well as the structure and dynamics of LiTFSI solutions in glyme solvents.^21–29^ Some of these works^26,28^ analyzed in detail the effects of correlations in ion motions, strongly influencing the charge transport and conductivity of concentrated electrolytes.^30^

In the past decade, several experimental works have investigated asymmetric perfluorinated sulfonimide anions,^31–34^ including also the structural isomer of TFSI, (fluorosulfonyl)­(pentafluoroethanesulfonyl)­imide (FPFSI).^31,33,34^ PEO-based electrolytes with LiFPFSI salt have shown improved stability and relatively high ionic conductivity,^33^ suggesting LiFPFSI as a promising alternative to LiTFSI. By contrast with the extensively studied TFSI, computational studies of the asymmetric FPFSI anion remain relatively scarce. Density functional theory calculations on perfluorinated anions and their binding to cations reported reduced interaction energies with Li^+^ and enhanced flexibility of asymmetric anions.^35^ In our recent work,^36^ we performed a detailed comparison of TFSI and FPFSI structures, their complexes with lithium cations, and vibrational spectra; the anions and ion aggregates were modeled in vacuum and in the effective solvent. We also used ab initio molecular dynamics (AIMD) simulations to study the LiTFSI and LiFPFSI solutions in tetraglyme (G4). Those calculations indicated a slightly weaker binding effect for the FPFSI anion and increased Li^+^ coordination to solvent molecules in the LiFPFSI electrolyte. However, the AIMD trajectory lengths were insufficient to estimate the dynamic properties of the system, including those governing ion transport, necessitating long classical MD simulations.

The goal of the present work was to use classical MD to compare the properties (structure, diffusion coefficients, conductivity, and transport numbers) of electrolytes containing two isomeric anions. To that end, we constructed a force field (FF) parametrization using a unified methodology for TFSI and FPFSI. Structural and transport properties were extracted from the MD trajectories for G4 electrolytes with three salt loadings. We discuss the similarities and differences between LiTFSI and LiFPFSI solutions in detail.

## 2 Computational Methods

NAMD v. 2.14 ^37^ was used in the MD simulations. Some additional quantum chemical (QC) calculations required for FF development were performed in Gaussian 09 rev. D.01.^38^

### 2.1 Force Field Parameterization

Bonded parameters for both anions and the G4 molecule were obtained using the Force Field Toolkit (FFtk)^39^ from the VMD modeling package.^40^ Geometry optimizations and Hessian matrix calculations, necessary to fit the force constants for bond stretching and angle bending, were performed at the MP2/aug-cc-pVDZ level. Torsional FF parameters were fitted to the dihedral angle scan profiles computed at the MP2/6–31+G* level. Partial atomic charges were based on the Merz–Kollman fit to the MP2/aug-cc-pVDZ electrostatic potential. Additional constraints were imposed when fitting the charges on the G4 molecule so that the total charge on the OCH_2_CH_2_ repeat unit and the sum of charges on terminal CH_3_ and OCH_3_ groups both equal zero. This allows reuse of the same set of charges in simulations for longer or shorter oligoglymes. Lennard–Jones (LJ) potential parameters for anions were taken from Köddermann’s work,^41^ and the same values were assumed for both isomers. The LJ parameters for G4 were adapted from the study on PEO,^42^ and the values for the Li^+^ cation were taken from ref 43. The initial nonpolarizable FF was then augmented by atomic polarizabilities. Polarization effects were accounted for via the Drude oscillator model;^44^ Drude particles were attached to all nonhydrogen atoms, except Li ions. The polarizabilities for TFSI were adapted from the APPLE&P parametrization.^45^ The same values were set for FPFSI, based on the QC results showing that the total polarizabilities of both isomers are almost the same.^36^ Atomic polarizabilities for G4 were taken from polarizable MD simulations of PEO.^46^ Throughout the work, we denote the oxygen atoms from G4 as O_g_ and the oxygen atoms from the anion as O_an_. Where needed for the FPFSI anion, the symbols O_SO_ and O_SF_ specify the oxygen atoms from the SO_2_ and SO_2_F groups, respectively.

Starting from this initial parametrization (hereafter denoted as FF0), we refined the parameters to improve the agreement between the FF-based and MP2 results. For this purpose, MP2/aug-cc-pVDZ calculations were performed to find the optimal geometries and binding energies of the Li-TFSI, Li-FPFSI, and [Li­(G4)]^+^ complexes (shown in Figure S1 in the Supporting Information). The same structures were then optimized in the FF, and the results were compared to the MP2 predictions. To test the parametrization in the simulations, for selected variants of the FF, we performed MD runs for the LiTFSI and LiFPFSI solutions in G4 with the Li/O_g_ ratios of 1:8 and 1:5; the simulation boxes contained 65:104 and 90:90 ion pairs/solvent molecules, respectively. The initial structures of the electrolytes were prepared in two ways: in one of them (systems *a*), cations, anions, and G4 molecules were randomly placed; in the other (systems *b*), Li^+^ cations were initially coordinated to solvent molecules. Test simulations were performed in the NpT (*p* = 1 atm, *T* = 303 K) and in the NVT (*T* = 303 K) ensemble; in the latter case, the volume of the system was set to reproduce the experimental densities of the LiTFSI 1:8 and 1:5 electrolytes.^9^ The length of the test trajectories was 15 ns (unless stated otherwise), with a 0.5 fs time step. All other details of the simulations were as in the production runs, as described at the end of this section.

Table S1 compares the QC and FF values for the Li-anion interaction energies and selected interatomic distances. The Li binding energies and most of the interatomic distances are fairly well reproduced by FF0. The exception is the Li–F distance in one of the Li-FPFSI geometries, becoming significantly shorter than the MP2 result (1.60 vs 1.98 Å). The test MD simulations revealed similarly short Li–F distances for both anions. Additionally, for both isomers, a similar effect was observed for the Li–N distances, resulting in improper Li-anion coordination (Figures S2–S3). To correct these artifacts, we modified the LJ parameters for the Li–F and Li–N interactions, using the NBFIX option (pair-specific LJ parameter override) implemented in NAMD. As seen in Table S1, this adjustment indeed corrects the Li–F distance, though at the price of a small decrease in the binding effect in the LiFPFSI pair. Consequently, the unphysical distances observed in MD simulations have been eliminated (Figures S2–S3). The fixed parametrization will be labeled FF1-base.

The Li–O distances and binding energies obtained from MP2 and FF calculations for the [Li­(G4)]^+^ complex are listed in Table S2 in the Supporting Information. The initial parametrization (FF0) overestimates the interaction energy, and accordingly, two of the Li–O distances are shortened by ∼0.2 Å with respect to the QC results. The binding of the lithium cation to tetraglyme is governed mostly by electrostatic interactions; therefore, adjustment of partial charges or polarizabilities of G4 atoms should, in principle, make it possible to modify the geometry of the complex and its binding energy. In Table S2, we report the results obtained for three FF modifications in which charges and/or polarizabilities of G4 atoms were scaled by a factor <1. Scaling only the charges improves the binding energy but has no effect on distances (parametrization FF0c). The latter can be improved when the polarizabilities are downscaled (in the parametrization FF1); in this case, the binding effect is still about 8 kcal/mol too strong. The best results for the optimized complex were obtained when scaling factors were applied for both charges and polarizabilities (FF1-sc). Therefore, FF1 and FF1-sc parameter sets were selected for the MD investigations. Note, however, that in both cases, the Li–O distances are still too small; despite many tests, we were not able to find a parametrization yielding a perfect agreement with MP2 results both for distances and for interaction energies, even with introducing NAMD NBFIX parameters to force the desired values of selected distances.

In Table S3, we compare the maxima of the radial distribution functions (RDFs) for Li–O_an_ and Li–O_g_ atom pairs and the coordination numbers (CNs) obtained for the 1:8 electrolytes of type *a* in the MD simulations using FF1 and FF1-sc parametrizations. Two general observations can be made from these data: Li–O_g_ distances are shorter than those for Li–O_an_ (1.91 Å vs 1.96–1.97 Å) regardless of the FF variant; in both FFs, the Li-G4 interactions are preferred over the Li-anion coordination, and this effect is larger in the FF1 parametrization. The effect of shorter Li–O_g_ contacts was observed in classical FFs^29^ and is in disagreement with AIMD simulations.^29,36^ Both parametrizations underestimate the density of the electrolyte by about 6%; the agreement to the experiment is better in FF1. Partially, this underestimate of the electrolyte density is related to the density of neat G4, which in FF1 simulations at 298 K is 0.987 g/cm^3^, that is, 2% lower than the experimental value 1.007 g/cm^3^.^47^

To assess the quality of structure reproduction by our FFs, the X-ray static structure factors (SSFs) for the 1:5 LiTFSI electrolyte were calculated using the ISAACS software^48^ and compared with the experimental data.^11^ We paid attention to the relative height of the two maxima at 1 and 1.5 Å^–1^, apparently related to the ratio of the Li–O_g_ and Li–O_an_ CNs. In the FF1-sc data, where the coordination to anions is the strongest, only the second maximum is pronounced, at slightly too low Q. On the other hand, at the beginning of the FF1 simulation for the type *b* electrolyte, where Li^+^ ions are almost exclusively complexed by solvent molecules, the first maximum is much higher than the second. The best agreement is observed for the equilibrated simulations in FF1, regardless of the initial structure (type *a* or *b*). It is worth noting that tests of parametrization variants in which the Li–O_g_ and Li–O_an_ distances were adjusted to better match AIMD simulations by applying NBFIX parameters resulted in structures having too small Li-G4 CNs and consequently in SSFs deviating from the measured data. Therefore, we decided to sacrifice the reproduction of the Li–O distances for the sake of better agreement of CNs and of the overall structure of the electrolyte with the scattering data. Accordingly, FF1 parametrization was selected for the main MD simulation in this work. The final values of the parameters are available in Appendix A in the Supporting Information, where we also summarized the sources of parameters (Table S6) and schematically depicted the workflow of the successive improvements of the parametrization (Scheme S1).

The test simulations indicated a technical problem related to the equilibration of the electrolyte. As seen in Figure S5, where we show the time evolution of the CNs, the final values of the CNs lie somewhere between the values obtained for the initial structures of types *a* and *b*, and the convergence to the equilibrated state of the system can be very slow. The changes are faster for the LiFPFSI electrolytes, and less concentrated systems relax faster than the 1:5 system, but for the latter, the equilibration times can reach 500 ns or more, and apparently neither type *a* or type *b* structures are a good starting point. For production MD simulations of multiple systems, the cost of equilibration would become prohibitive. However, Figure S5 suggests that the initial coordination of Li^+^ ions should be between structures *a* and *b*, and such a setup was used to prepare the main simulations.

### 2.2 Simulation Details

The systems investigated in the classical MD simulations were the LiTFSI and LiFPFSI solutions in G4 at three Li/O_g_ ratios: 1:20, 1:8, and 1:5; the latter concentration corresponds to an equimolar salt/solvent composition. To improve sampling, we simulated 5 independent replicas of each system at the 1:20 or 1:8 ratios and 10 replicas for the most concentrated (and therefore the most viscous) 1:5 electrolytes. Table 1 shows the number of ions and solvent molecules in each system. The initial structures were built using Packmol software.^49^ To shorten the equilibration time, half of the Li^+^ ions were introduced as [Li­(G4)]^+^ complexes, whereas the other half was placed randomly in the simulation box.

**Table 1:** Compositions of Studied Systems and the Densities Used in the NVT Simulations at *T* = 303 K

Li/O_g_	# of ion pairs	# of G4 molecules	*a*, Å	density, g/cm^3^
1:20	30	120	36.966	1.16
1:8	65	104	37.495	1.316
1:5	90	90	37.885	1.400

Simulations were performed in the NVT ensemble using Langevin dynamics at *T* = 303 K and the volume of the periodic cell set to reproduce the experimental densities of LiTFSI/G4 electrolytes.^9^ The first 10 ns of the trajectory were simulated with the 0.5 fs time step to prevent the instabilities caused by fast Li^+^ motions; after that time, the time step was increased to 1 fs. The lengths of the recorded trajectories were 450 ns for the 1:5 systems and 400 ns for the less concentrated electrolytes. Structural data were averaged over the last 20 ns of simulations; we checked that the results did not differ significantly when the last 40 ns were used, indicating that the structures of the electrolytes reached an equilibration stage. Longer parts of trajectories are required in calculations of dynamical properties (to capture the diffusive regime of the system), and the last 250 ns were used for this purpose. In all cases, the results were averaged over all of the simulated replicas.

## 3 Results and Discussion

### 3.1 Structure of the Electrolytes

The structure of the simulated solutions was assessed through the analysis of RDFs for selected atom pairs and coordination numbers of Li^+^ ions. In Figure S6, we show the changes in the Li–O CNs during the simulations. Even for the most concentrated 1:5 electrolytes, after 250 ns, there are no systematic changes of the average values; for the systems with lower salt loading, the final values were reached even faster.

The RDFs for the Li–O pairs are shown in Figure 1. Regardless of the anion, the distance of the first maximum in the Li–O_g_ RDF increases slightly with salt concentration from 1.89 to 1.91 Å. The distances to the oxygen atoms of the anion are longer and essentially independent of the electrolyte concentration. The Li–O_an_ (LiTFSI electrolytes) and Li–O_SO_ distances (LiFPFSI solutions) are 1.96 and 1.95 Å, respectively. In the latter electrolytes, the Li–O_SF_ distances are about 1.97 Å, indicating that the Li^+^ interaction with the SO_2_F group is somewhat weaker; this result was also obtained earlier in the AIMD simulations.^36^ As stated in the preceding section, the Li–O_g_ distance is shorter than that obtained from AIMD results,^29,36^ yet, as shown below, this does not lead to an overestimate of the Li–O_g_ CN. Indeed, our results are in agreement with the simulations of LiTFSI/G4 electrolytes based on the interaction potentials obtained from the fits to the neutron diffraction data using empirical potential structure refinement (EPSR).^12^ In the latter work, the Li–O_g_ correlations produced peaks at 1.90 Å, whereas Li–O_an_ correlations were observed at 2.0 Å; therefore, the relation of Li–O distances in our simulations matches the structural data extracted from diffraction experiments.

**Figure 1:**
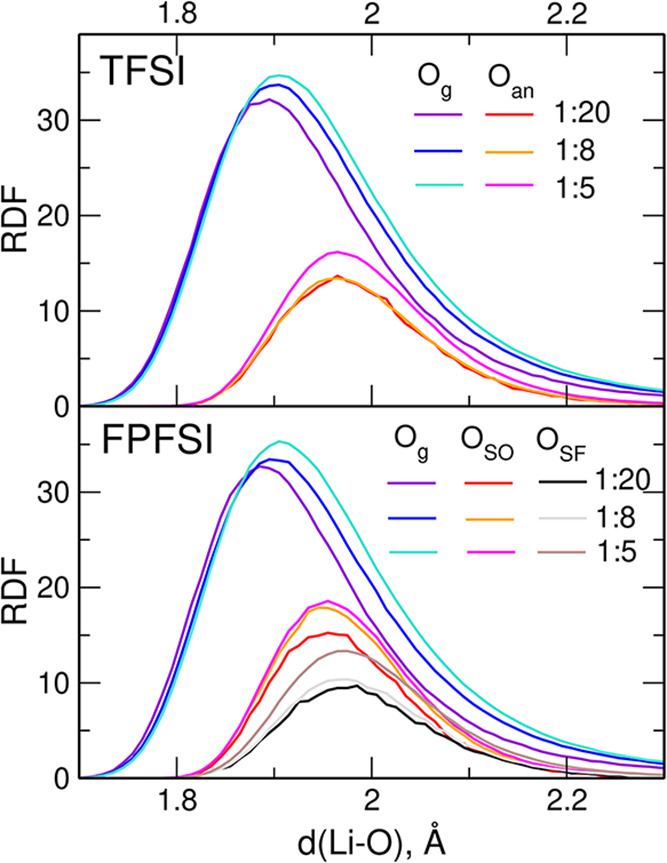
RDFs for Li–O atom pairs in the LiTFSI and LiFPFSI solutions in G4 at different Li/O_g_ ratios.

The coordination numbers of Li cations are very similar in both types of electrolytes; therefore, in Figure 2, we show the concentration dependence of the running CNs (integrated RDFs) only for LiFPFSI solutions. A comparison of CNs for the 1:5 LiTFSI and LiFPFSI electrolytes is presented in Figure S7. In Table S4 in the Supporting Information, we collected the values of the average Li–O CNs integrated to the 3 Å distance from the cation. As expected, increasing the salt content from a 1:20 to 1:5 ratio promotes Li–O_an_ interactions (increase of the CN from 0.27 to 0.99) at the expense of Li–O_g_ coordination (decreasing from 4.30 to 3.56). The number of coordinating O_SO_ oxygens is always larger than the CN for O_SF_ atoms, confirming the conclusion of a weaker Li–O_SF_ interaction, as suggested by the RDF plots. As seen in Figure S7 and Table S4, the differences in CNs between LiTFSI and LiFPFSI solutions are small and amount to less than 0.05 at the highest salt loading. Finally, we note that the total Li–O coordination number is in the range of 4.55–4.65 and is fairly independent of the salt content; that is, the decrease in coordination to solvent molecules is compensated by Li^+^ interactions with anions. Although the Li–O_g_ distances are shorter than the Li–O_an_ separation, the Li–O_g_ CNs are lower than those reported in most of the works using classical MD.^21,25,27^ Our values are closest to ref 22. In particular, the Li–O_g_ CN obtained for LiTFSI/G4 in the FF derived from EPSR to neutron diffraction data was even lower (∼2.3), and the total Li–O CN amounted to 4.^12^ Therefore, we conclude that our simulations provide a fairly good description of the structure of the electrolyte.

**Figure 2:**
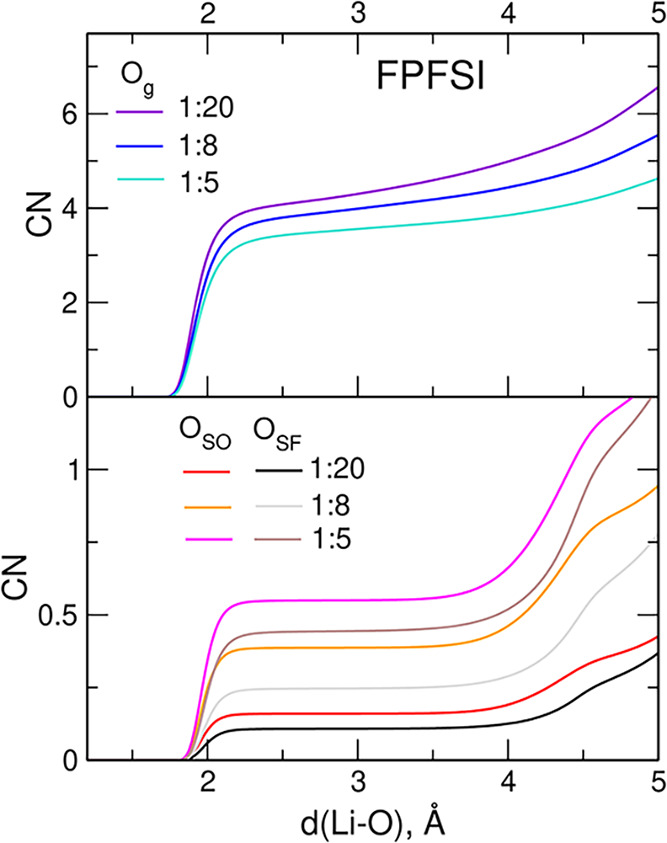
Integrated RDFs for Li–O atom pairs in the LiFPFSI solutions in G4 at different Li/O_g_ ratios.

In Figure S8, we display the Li–F RDFs for both types of solutions at the 1:5 Li/O_g_ ratio. For both anions, the first notable maximum appears above 4 Å and is located at the shortest distance of 4.2 Å for the F atoms from the SO_2_F group of FPFSI. In this case, there are nonzero values of the RDF at 2 Å, showing a tiny amount of Li–F interactions in the LiFPFSI solutions (as expected from the QC calculations of ref 36). Nevertheless, the Li–F CN integrated up to 3 Å is only 0.013; hence, this kind of Li^+^ coordination is negligible.

CNs provide information about average coordination only. To gain more insight into the interactions of Li cations with solvent molecules and salt anions, we analyzed the Li^+^ speciation, that is, the abundance of different coordination shells. The results are presented in Figure 3, where we used a threshold of 3 Å to count the O atoms that coordinate the cation.

**Figure 3:**
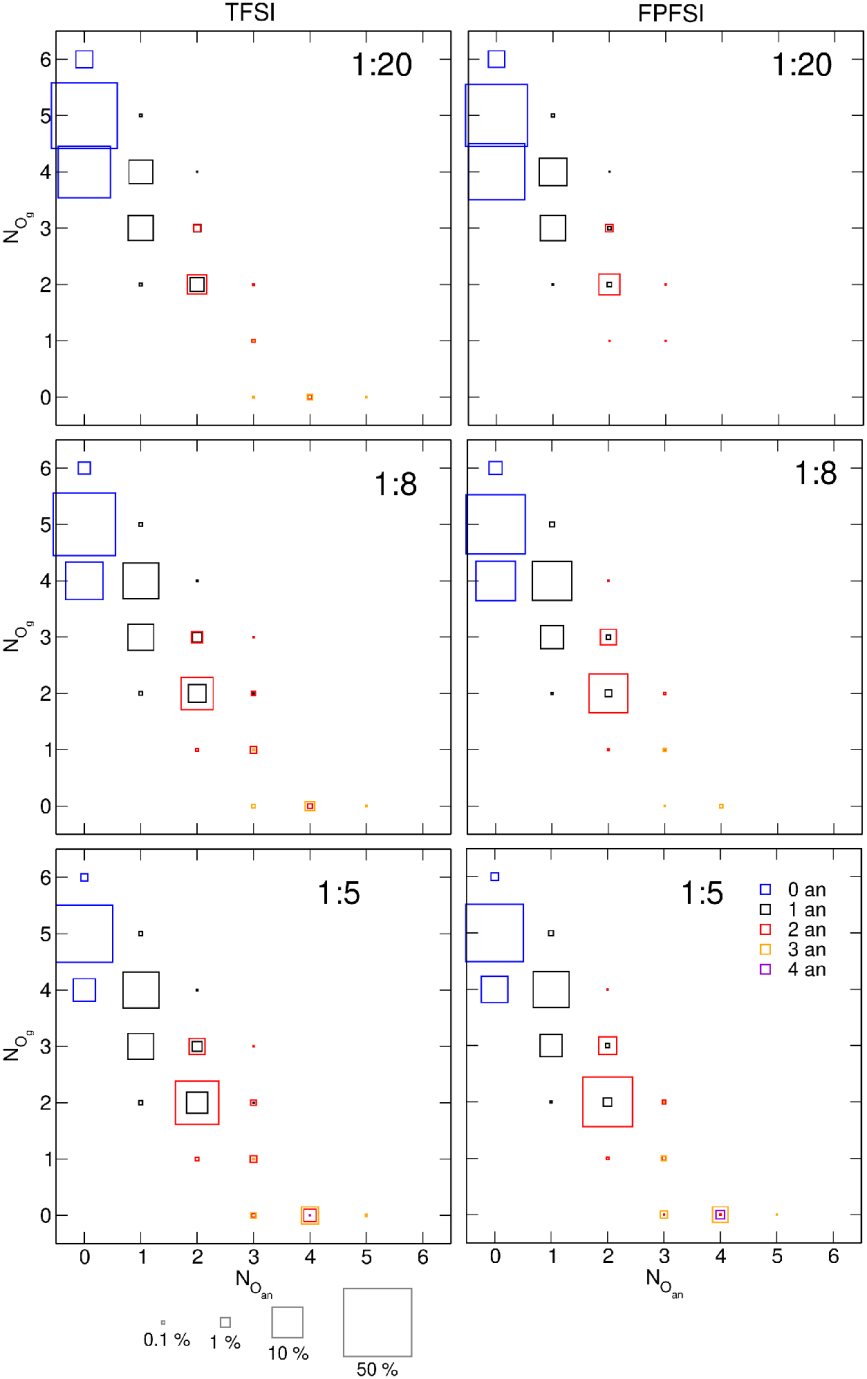
Abundance of different coordination environments in the LiTFSI and LiFPFSI electrolytes at different Li/O_g_ ratios. Areas of squares are proportional to the abundance.

For both salts, the dominant type of Li binding is coordination solely to solvent molecules. At the lowest concentration, it amounts to 79% and, although it decreases to 42 and 45% in the 1:5 LiTFSI and LiFPFSI electrolytes, respectively, it is still the largest contribution. The most probable motif is always Li^+^ interaction with 5 O_g_ atoms. In the 1:20 electrolytes, coordination to 4 O_g_ atoms is also quite frequent, but it declines fast with salt concentration (e.g., from 34% in the 1:20 LiFPFSI to 8% in the 1:5 LiFPFSI electrolyte). Conversely, Li^+^ coordination to 5 solvent oxygens is only weakly concentration-dependent (42 and 36% in the 1:20 and 1:5 LiFPFSI systems, respectively), suggesting that the structure with the G4 molecule wrapped around the Li cation is particularly stable.

The probability of Li coordination to a single O_an_ atom (typically with 3 or 4 O_g_ atoms) does not change much with the salt content: 12.9, 21.5, and 21.7% for LiTFSI at 1:20, 1:8, and 1:5 Li/O_g_ ratio, respectively; the corresponding values for LiFPFSI electrolytes read 15.3, 22.7, and 19.6%. At the 1:20 ratio, contributions from 1O_an_3O_g_ and 1O_an_4O_g_ solvation shells are similar, and the abundance of the latter increases with salt concentration. Salt loading largely influences the abundance of configurations with two O_an_ atoms: from 7.1 and 5.7% in the 1:20 LiTFSI and LiFPFSI electrolytes, respectively, to 29.3 and 31.2% in the 1:5 systems. It is noticeable in Figure 3 that the bidentate coordination is less preferred by FPFSI anions. At the 1:5 Li/O_g_ ratio, the total probability of the 2O_an_2O_g_ and 2O_an_3O_g_ shells (where both O_an_ atoms are from the same anion) is 5.9% in the LiTFSI electrolyte (closely to the 5.5% for bidentate TFSI binding found in ref 27) but only 0.9% in the LiFPFSI system. The abundance of Li^+^ coordination shells involving more than 2O_an_ atoms is small, even in the most concentrated solutions (and it is larger in the LiTFSI electrolytes).

The probability of Li^+^ binding solely to G4 molecules in the LiFPFSI electrolytes is slightly higher than that in the LiTFSI solutions. With an observation that the monodentate anion binding is more abundant in the former, these data suggest that the Li-FPFSI interactions are a little weaker than the binding of the Li^+^ cation to TFSI anions, as already suggested by the QC calculations and AIMD simulations in ref 36 and a previous QC study on asymmetric fluorinated anions.^35^

In order to obtain more details on the binding pattern of G4 molecules, we calculated Venn diagrams, showing the percentage of solvent molecules engaging a specified subset of O_g_ atoms in interactions with Li^+^ cations (the Li–O_g_ distance threshold was 3 Å). There are only small differences between LiTFSI and LiFPFSI electrolytes; therefore, in Figure 4, we display the results for LiFPFSI solutions, and the data for LiTFSI systems are available in Figure S9 in the Supporting Information. The graphs were symmetrized with respect to the equivalent atoms of G4.

**Figure 4:**
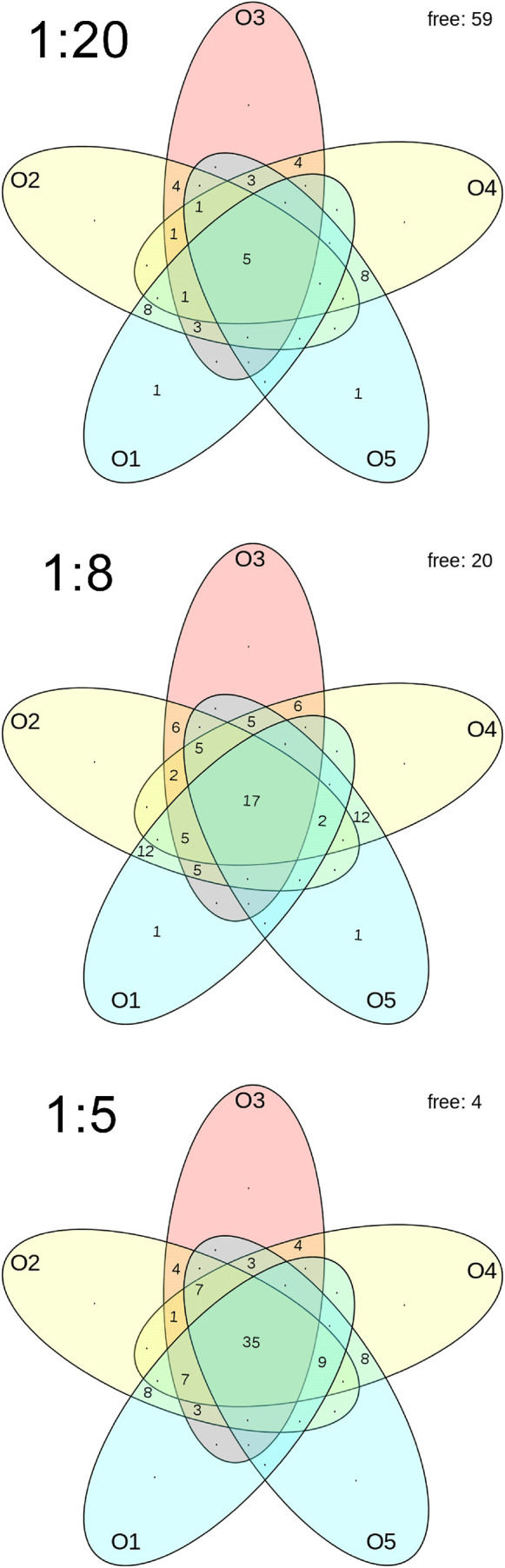
Venn diagrams showing the connectivity between the Li^+^ ions and O_g_ atoms in LiFPFSI electrolytes. Values smaller than 1% are displayed as dots.

At the 1:20 Li/O_g_ ratio, the number of free solvent molecules reaches 60% and decreases rapidly with salt content; in the 1:5 systems, only 5% of G4 molecules are not interacting with Li cations. At low salt concentration, the most probable is the Li^+^ coordination via two O_g_ atoms (22–24%), and only 5–7% of G4 molecules engage all five oxygen atoms in interactions with the cation (or cations). The probability of 2-fold coordination remains unchanged in the most concentrated electrolytes but equally probable are the interactions through four O_g_ atoms (23–25%), and the 5-fold coordination engaging all of the O_g_ atoms of G4 is dominating (35%). Finally, we should note that when a solvent molecule coordinates Li^+^ via more than one O_g_ atom, in more than 85% of cases, the interacting atoms are the consecutive atoms along the molecule. While the amount of free glyme molecules in the 1:5 LiTFSI electrolyte agrees with analogous analysis in ref 27, the previous study predicted a larger abundance of 5-fold Li^+^ coordination: 82% vs our result of 34%. Nevertheless, as we will see in the following section, the amount of four- and 5-fold coordinating G4 molecules is sufficient to form stable [Li­(G4)]^+^ complexes.

We also examined the conformations of salt anions in the electrolyte and their dependence on the salt concentration. Figure 5 shows the distributions of selected dihedral angles defining the geometry of the anion. According to the plots, TFSI anions are in the *gauche* (CSSC angles of about 40° or 90°) and *trans* conformations (CSSC angle close to 170°), whereas the dihedral angles for FPFSI anions correspond to several *gauche* geometries (Cf. Tables S2 and S3 in the Supporting Information of ref 36). Changes in the above distributions in electrolytes with different salt concentrations are displayed in Figures S10 and S11. Although the changes are modest, two dihedrals, namely the CSSC angle for the TFSI anions and the SNSC angle of the FPFSI anions, seem to be the most sensitive to the electrolyte concentration. Therefore, in Figure 6, we present the distributions of these angles obtained for the 1:5 systems separately for free anions and those interacting with Li^+^ cations with one or two O_an_ atoms. The changes seen in Figure S10 for the CSSC angle (increase of values close to 0° and 100–110°) result from an increasing probability of monodentate anion coordination. The amount of anions involved in bidentate coordination is not sufficient to significantly affect the distribution, even though such a coordination changes the CSSC distribution by increasing the probability of the *trans* conformation of the anion, that is, angles close to 170°. For FPFSI anions, coordination shifts the maximum in the θ­(SNSC) distribution from 50 to 70° and gives rise to a secondary maximum at about 130°. In particular, the distribution of the SNSC dihedral observed in Figure 6 for FPFSI interacting through two oxygens corresponds to a bidentate Li^+^ coordination using O_SO_ and O_SF_ atoms.

**Figure 5:**
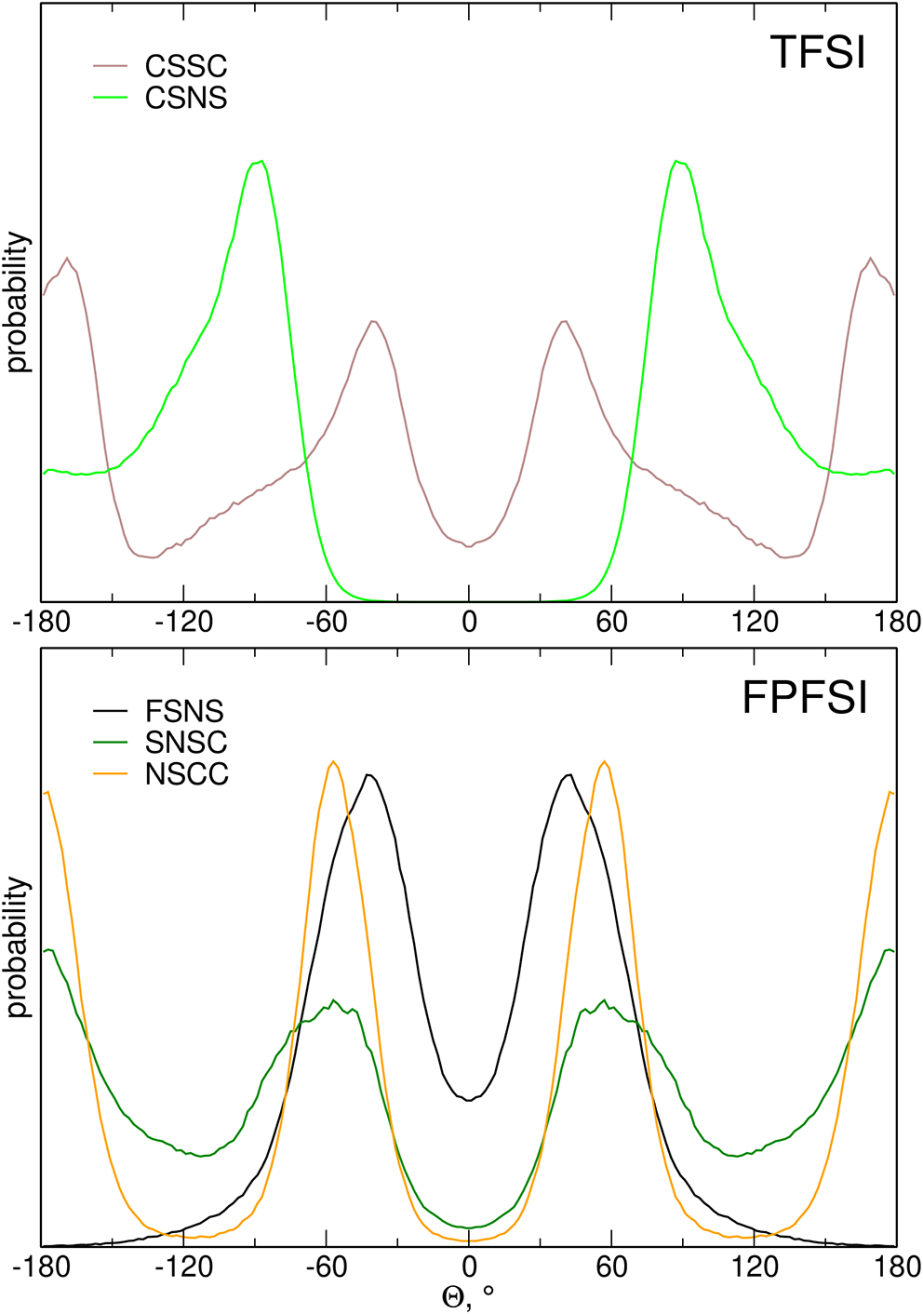
Distributions of the values of dihedral angles in TFSI and FPFSI anions in the LiTFSI/G4 and LiFPFSI/G4 electrolytes at Li/O_g_ = 1:20.

**Figure 6:**
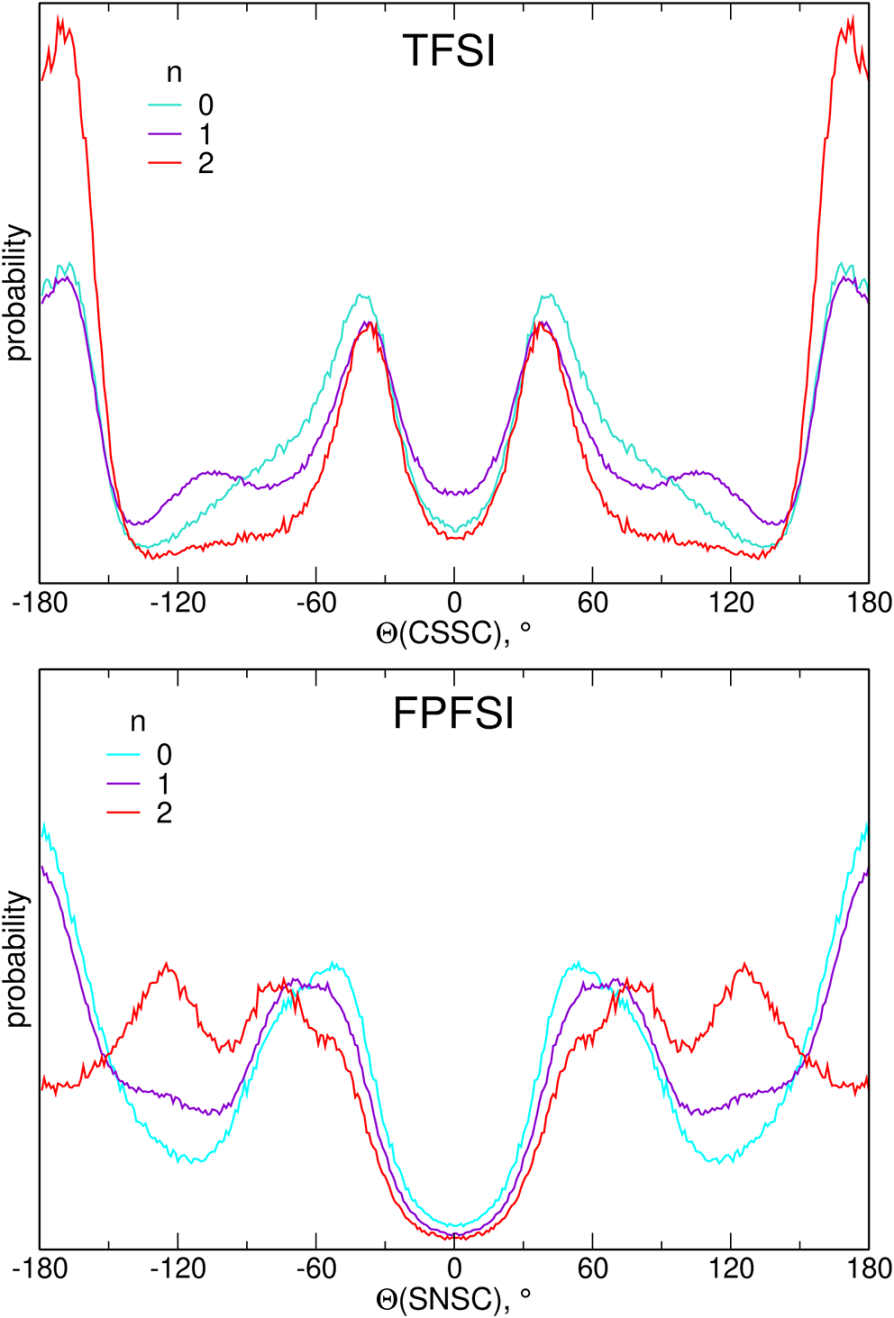
Distributions of the values of selected angles in TFSI and FPFSI anions in different coordination environments in the LiTFSI/G4 and LiFPFSI/G4 electrolytes at a Li/O_g_ = 1:5. The number of O atoms of the anion coordinated to the Li^+^ cation(s) is *n*.

### 3.2 Dynamics

To obtain some estimates on the time scale of Li^+^ exchange events between anions or solvent molecules, we computed the autocorrelation functions (ACFs) of the Li–O residence time

1 $$C_{\mathrm{Li}-O}\left(t\right)=\frac{\left\langle H_{\mathrm{ij}}\left(t\right)H_{\mathrm{ij}}\left(0\right)\right\rangle }{\left\langle H_{\mathrm{ij}}\left(0\right)H_{\mathrm{ij}}\left(0\right)\right\rangle }$$

where *H*_*ij*_(*t*) = 1 if at time *t*, the distance between the *i*th Li^+^ cation and the *j*th O atom is smaller than a threshold value of 3 Å or *H*_*ij*_ = 0 otherwise. We further defined Li-solvent and Li-anion residence-time ACFs *C*_Li‑G4_ and *C*_Li‑an_ using *H*_*ij*_(t) = 1 if any of the oxygen atoms from the anion/solvent molecule *j* is coordinated to the cation *i* (that is, if it falls within the threshold distance of 3 Å). For a quantitative description, the stretched exponential functions exp­[-(*t*/τ)^α^] were fitted to the *C*(*t*) functions, yielding the oxygen atom, anion, or solvent residence times τ.

The sample plots of ACFs and exponential fits for the 1:5 electrolytes are shown in Figure 7; the results for other systems are available as Figures S12–S13 in the Supporting Information. Parameters of the fits are collected in Table S5, and the dependence of the residence times τ on the electrolyte concentration is presented in Figure 8.

**Figure 7:**
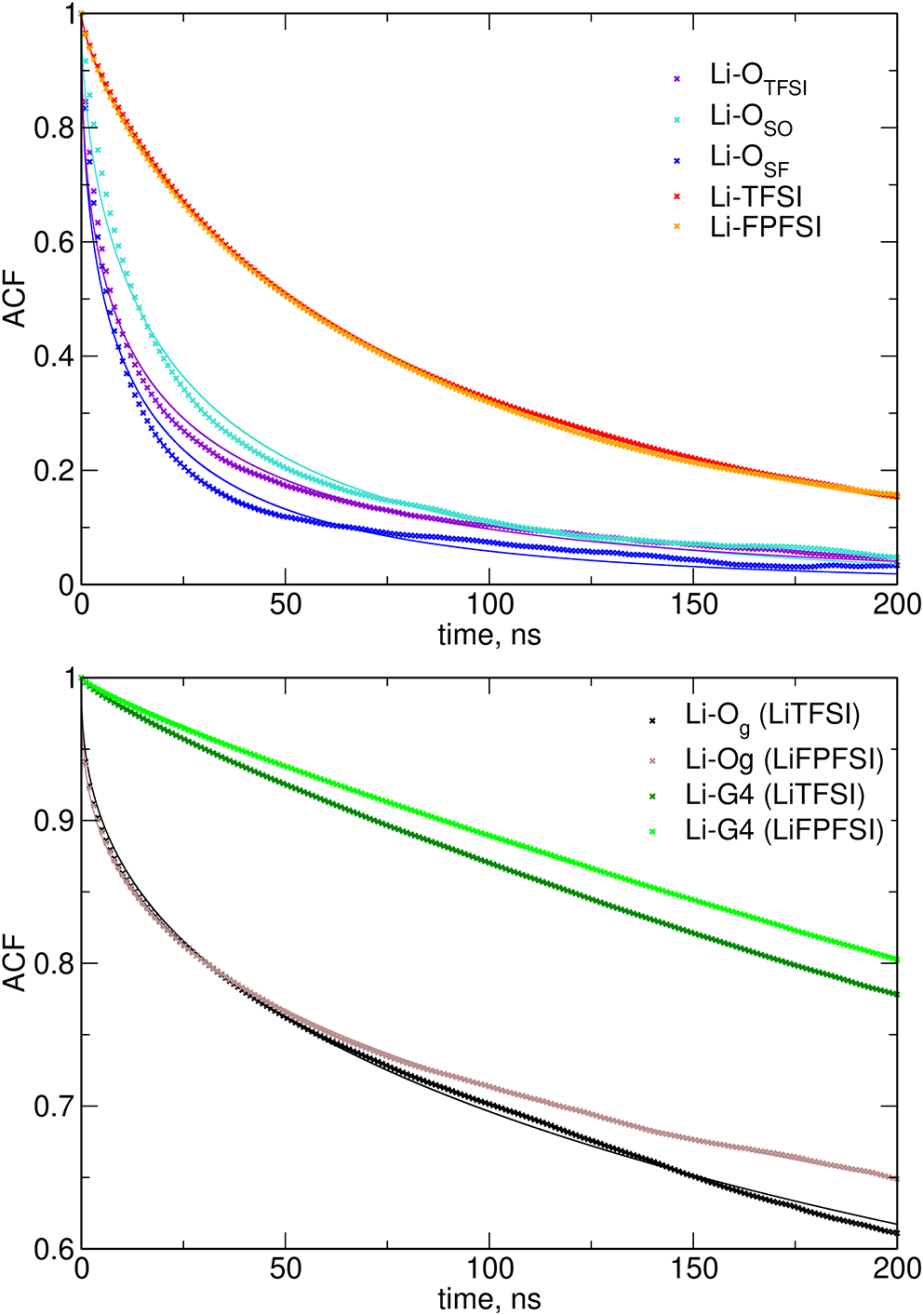
Autocorrelation functions for Li^+^ interactions with O atoms, anions, or solvent molecules in the Li/O_g_ = 1:5 LiTFSI and LiFPFSI electrolytes. Lines are fitted to the data.

**Figure 8:**
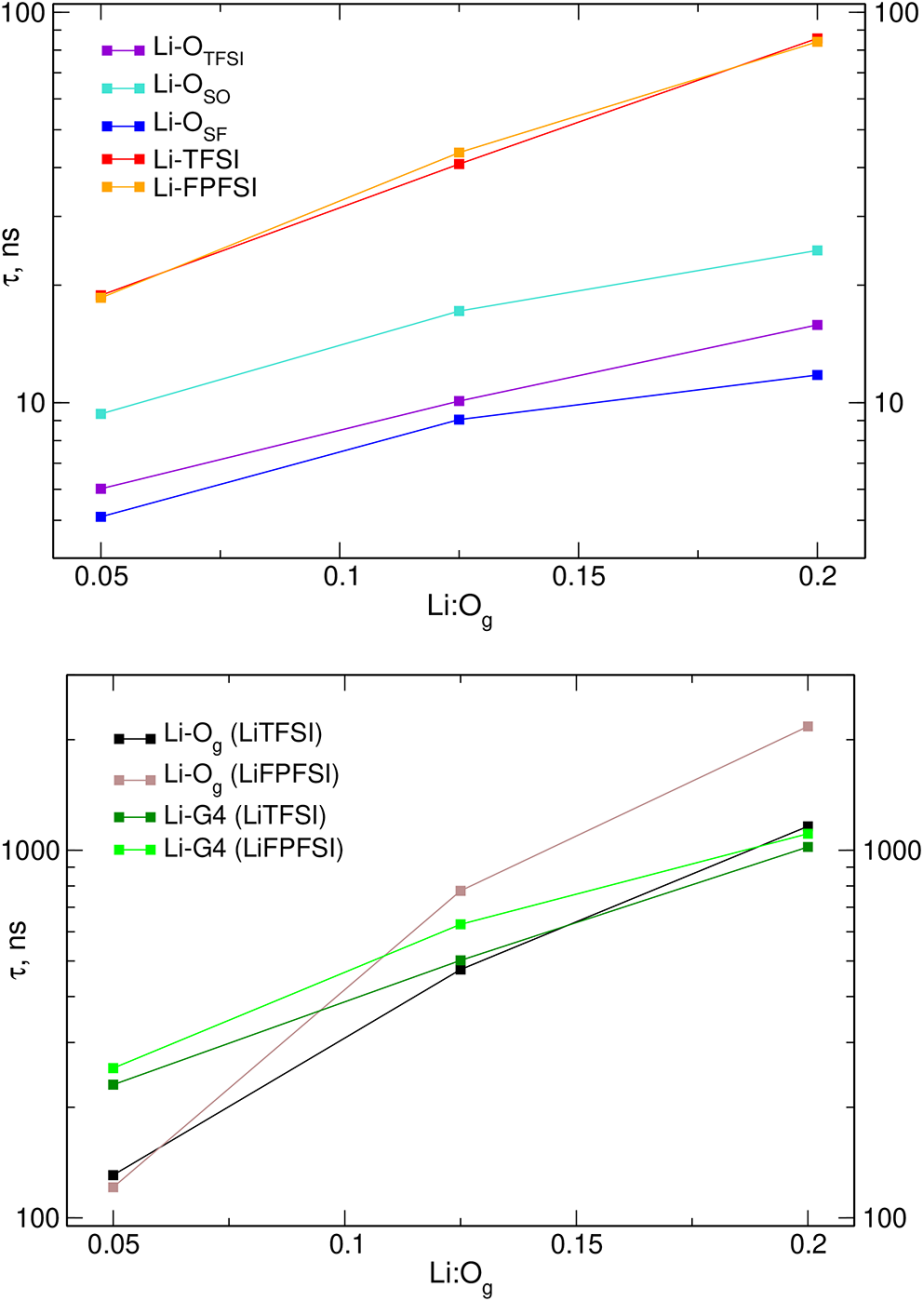
Residence times τ for Li^+^ interactions with O atoms, anions, or solvent molecules in LiTFSI and LiFPFSI electrolytes in G4 at different Li/O_g_ ratios.

There are visible differences when ACFs for individual Li–O_an_ interactions are considered: residence times for O_SO_ atoms are about two times larger than those for O_SF_ oxygens, and the residence times for the Li–O_TFSI_ interactions have intermediate values between the two above. Therefore, the Li–O_SF_ interactions are the most easily broken (in accord with earlier indications that bonding to O_SF_ atoms is weaker than that to O_SO_ oxygens), and the longest-lasting are the bonds to O_SO_ atoms. Nevertheless, the overall Li-anion residence times are practically the same for both anions and 2–7 times larger than the τ values for individual O_an_ atoms. Although the Li^+^ contacts with the oxygen atoms of the ion can be broken quite fast, the cation remains in contact with the anion, exchanging only the individual coordinating atoms.

Residence times for Li-solvent interactions are 1–2 orders of magnitude longer than those for anions. We can note that the Li–O_g_ and Li-G4 ACFs do not reach the zero value within the time of MD simulations; accordingly, the estimated residence times are supposed to carry large uncertainties; nevertheless, they can be used for the analysis of trends. The τ values for the 1:8 and 1:5 electrolytes can be confusing because for Li–O_g_, they are longer than for Li-solvent, despite the interactions with the whole G4 molecule having to last longer than the interactions with its individual atoms. The reason for this dependence originates from different values of the stretching factor α in the exponent, which are 0.33–0.41 for Li–O_g_ interactions and 0.85–0.96 for Li-G4. Therefore, τ values can be safely compared only for the same type of interaction. As readily seen in Figures 7 and S12–S13, the Li-G4 ACFs decay more slowly than the ACFs for Li–O_g_, as expected. It can be seen that τ_Li‑G4_ in LiFPFSI electrolytes is always longer than in LiTFSI solutions, and a similar trend is observed for τ_Li‑Og_ (except for the 1:20 electrolytes, where the values are quite similar). Apparently, the anion of the salt influences the time scale of Li-solvent interactions, as FPFSI anions promote Li-G4 complexes with lifetimes longer than those in the LiTFSI solutions.

For all types of interactions, residence times increase with salt concentration, and in the 1:5 electrolytes, they are typically about 4 times larger than in the 1:20 systems, and this can be related to the increasing viscosity. Notably, the residence times for Li-G4 interactions approach the microsecond scale at the 1:5 Li/O_g_ ratio, confirming the stability of solvates in the equimolar salt solutions in tetraglyme. These findings are consistent with those from earlier MD studies of LiTFSI/G4 electrolytes. In the simulations of refs 25 and 26 employing polarizable APPLE&P force field, an order of magnitude increase of Li–O_g_ residence times was found at 373 K when the Li/O_g_ ratio increased from 1:20 to 1:5. At 303 K in the 1:5 electrolyte, the Li–O_an_ residence time was 7 ns;^25^ our simulations yielded 16 ns in reasonable agreement. The nonpolarizable MD simulations in ref 24 were performed at 503 K, so that the direct comparison of data is not possible, but their results (τ_Li‑G4_ = 87 ns and τ_Li‑an_ = 2.5 ns) indicate that anion exchange around Li^+^ cation is much faster than the exchange of solvent, owing to the stability of [Li­(G4)]^+^ solvates.

Based on the recorded MD trajectories, we calculated estimates of parameters relevant to the ion transport in the electrolyte. The diffusion coefficient of species *i* (ion or G4 molecule) was obtained from the slope of its mean square displacement (MSD) dependence on time

2 $$D_{i}=\lim_{t\to \infty }\frac{1}{6t}\left\langle {\left[R_{i}\left(t\right)-R_{i}\left(0\right)\right]}^{2}\right\rangle$$

The conductivity of the electrolyte was estimated from the collective displacements of ions using the Einstein formula as

3 $$\sigma =\lim_{t\to \infty }\frac{e^{2}}{6\mathrm{tV}k_{B}T}\sum_{i,j}z_{i}z_{j}\left\langle \left[R_{i}\left(t\right)-R_{i}\left(0\right)\right]\left[R_{j}\left(t\right)-R_{j}\left(0\right)\right]\right\rangle$$

In the above formulas, *t* stands for time, *V* is the volume of the system, *k*_B_ is the Boltzmann constant, *T* is the temperature, *e* is the elementary charge, *z*_*i*_ and *z*_*j*_ are the charges of the ions *i* and *j*, **R**_*i*_ (*t*) is the position of the *i*th ion at time *t*, and the brackets ⟨⟩ denote the ensemble average. The results were averaged over all possible choices of time interval within the trajectory; diffusion coefficients and conductivities were calculated from the linear part of the plot. Sample plots of averaged MSDs are shown in Figures S14 and S15 in the Supporting Information.

The calculated diffusion coefficients of the ions and solvent molecules and the conductivities of the electrolytes are collected in Table 2. The mobilities of solvent molecules and ions are on the order of 10^–8^ cm^2^/s and decrease with salt content. Values of 1.2–1.3 × 10^–8^ cm^2^/s calculated for the 1:5 LiTFSI electrolyte are 1 order of magnitude smaller than the measured diffusion coefficients.^30^ The source of this difference is the underestimated *D*_G4_ of the neat solvent; test MD simulations at 298 K give ∼6 × 10^–7^ cm^2^/s, whereas the experimental value reads 3.4 × 10^–6^ cm^2^/s.^50^ In effect, the calculated diffusion coefficients in the electrolytes (as well as the conductivities) are too low. However, because our aim was to compare two salts, we will analyze the transport data, paying attention to the trends rather than to individual values.

**Table 2:** Conductivities, Diffusion Coefficients, Cation Transport, and Transference Numbers Obtained from MD Simulations for LiTFSI/LiFPFSI Electrolytes in Tetraglyme

	1:20		1:8		1:5	
electrolyte	TFSI	FPFSI	TFSI	FPFSI	TFSI	FPFSI
σ, S/m	0.030	0.043	0.016	0.027	0.014	0.020
*D*_G4_, cm^2^/s	1.8 × 10^–7^	1.8 × 10^–7^	3.2 × 10^–8^	3.8 × 10^–8^	1.5 × 10^–8^	1.6 × 10^–8^
*D*_+_, cm^2^/s	9.7 × 10^–8^	9.0 × 10^–8^	2.4 × 10^–8^	2.7 × 10^–8^	1.2 × 10^–8^	1.3 × 10^–8^
*D*_–_, cm^2^/s	1.2 × 10^–7^	1.2 × 10^–7^	2.7 × 10^–8^	2.8 × 10^–8^	1.3 × 10^–8^	1.4 × 10^–8^
*t*_+_^*D*^	0.45	0.43	0.47	0.49	0.48	0.49
*t*_+_^*c*^	0.38	0.47	0.49	0.50	0.52	0.54
*t*_+_^*abc*^	0.57	0.48	0.33	0.32	0.23	0.24

With the exception of the least concentrated 1:20 electrolyte, diffusion coefficients in LiFPFSI solutions seem systematically slightly larger than those in the corresponding LiTFSI electrolytes, but the difference is rather small. As seen in Table 2 and in Figure 9, the diffusion coefficient of the solvent *D*_G4_ is larger than the coefficient for Li^+^ ions *D*_+_, but both values tend to converge with increasing salt concentration. The *D*_G4_/*D*_+_ ratio in the 1:20 electrolytes is about 1.9–2.0 and decreases to 1.2 at the 1:5 Li/O_g_ ratio. It is another indication of a special behavior of salt solutions in G4. At low concentrations, the system is a salt solution in a molecular liquid, with *D*_solvent_ > *D*_+_. In the equimolar system, [Li­(G4)]^+^ solvates are formed, salt cations and solvent molecules codiffuse, leading to *D*_G4_ ≈ *D*_+_, and the solution behaves as a solvate ionic liquid.

**Figure 9:**
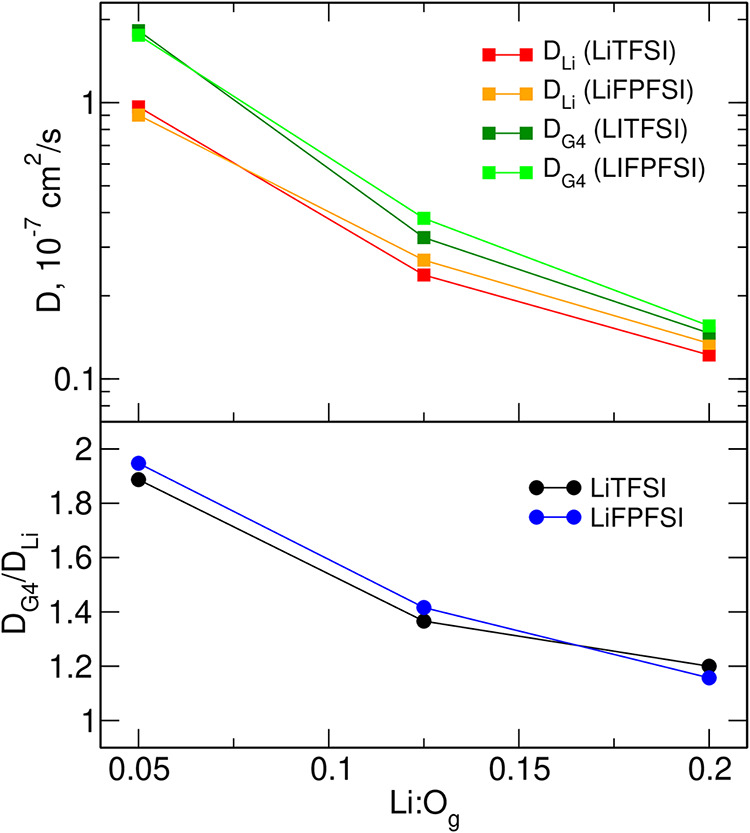
Diffusion coefficients of Li^+^ cations and G4 molecules at different electrolyte concentrations (top) and the *D*_G4_/*D*_Li_ ratio (bottom).

For both types of salt, the conductivity of the electrolyte decreases with salt concentration, and it is notably (40–70%) larger for LiFPFSI-based electrolytes, despite the diffusion coefficients of ions in LiTFSI and LiFPFSI solutions being very similar. This interesting observation suggests that the correlations between motions of ions play an important role in the electrolytes studied here. To analyze this effect in more detail, we decomposed the sum in eq 3 into different contributions according to the indices *i* and *j*

4 $$\sigma =\sigma_{+}+\sigma_{-}+\sigma_{++}+\sigma_{--}-2\sigma_{+-}$$

The diagonal (*i* = *j*) terms σ_+_ and σ_–_ are related to the self-diffusion of cations and anions, respectively, and are proportional to *D*_+_ and *D*_–_. The off-diagonal (*i* ≠ *j*) terms arise from correlations between motions of different ions: cation–cation (σ_++_), anion–anion (σ_––_), and cation–anion (σ_+–_). The σ_+_ and σ_–_ terms are usually referred to as “self” contributions, whereas the σ_++_ and σ_––_ are called “distinct” contributions. For consistency with the notation commonly applied for Onsager coefficients, we used the −2 factor before the last term.^28,30^ Contributions to the conductivity were calculated by using the center-of-mass (COM) reference frame, as typical in MD simulations. Note that the experimental transference numbers in ref 30, to which we will compare our data, were converted to the COM frame.

The partition of the total conductivity into different contributions is shown in Figure 10. It is convenient to recall here the basic difference between ionic liquids and salt solutions.^51^ In a typical salt solution in a molecular liquid, all off-diagonal correlation terms σ_++_, σ_––_, and −2σ_+–_ are negative, and thus, correlations reduce the total conductivity. In ionic liquids (or, more generally, in molten salts), the cation–anion correlation term −2σ_+–_ is positive, contributing constructively to the conductivity. This effect originates from conservation of the total momentum of the IL, imposing anticorrelated motions of ions of opposite charges.^51^ In salt solutions in molecular liquids, this restriction does not apply because momentum can be balanced by the motions of the neutral solvent molecules.

**Figure 10:**
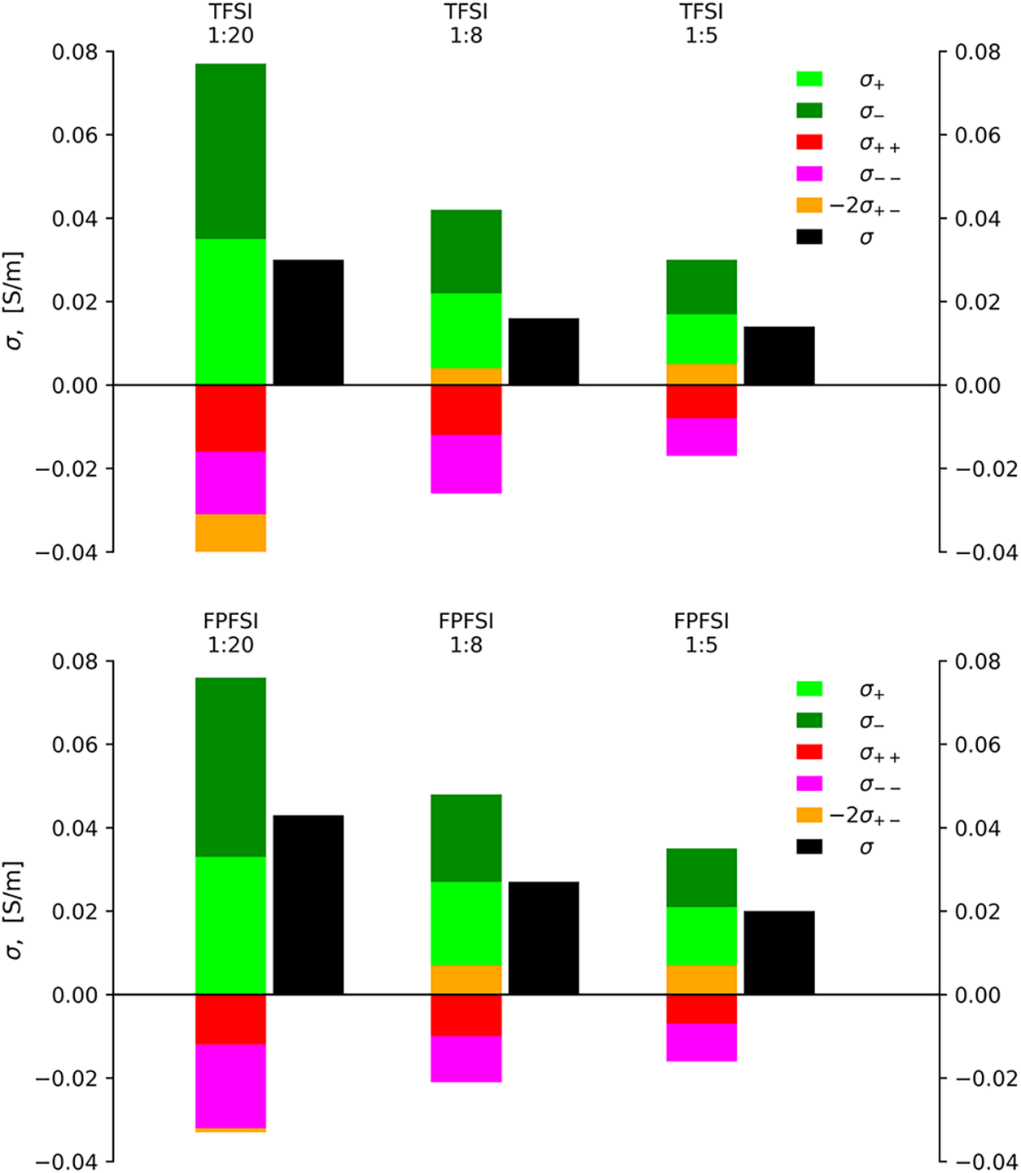
Contributions (according to eq 4) to the total conductivity σ of the LiTFSI and LiFPFSI solutions in G4 at different Li/O_g_ ratios.

As seen in Figure 10, at a low salt concentration (1:20 Li/O_g_ ratio), the electrolyte is a salt solution in a liquid, with all correlation contributions being negative (although the σ_+‑_ contribution in the LiFPFSI solution is close to 0). At higher concentrations, the −2σ_+–_ term becomes positive, as expected in ILs, in accord with a picture of the equimolar electrolyte, with complex [Li­(G4)]^+^ cations and free TFSI/FPFSI anions forming a solvate ionic liquid. The −2σ_+–_ contribution has larger (more positive) values in LiFPFSI electrolytes, leading to higher total conductivities. Therefore, the conductivity enhancement in LiFPFSI solutions can be attributed to a more favorable balance of ion–ion correlations. The studied systems represent a notable case in which weaker cation–anion interactions for the LiFPFSI salt counterintuitively increase the degree of cation–anion correlations in the electrolyte. However, the effect is indirect: weaker Li-FPFSI interactions strengthen Li-solvent binding, bringing the LiFPFSI electrolytes closer to an “ideal” SIL with substantial anticorrelation in cation–anion movements.

As discussed earlier, our diffusion coefficients are underestimated; accordingly, estimates of the conductivity are too small, e.g., the σ value calculated for an equimolar LiTFSI/G4 solution is 0.0144 S/m, about an order of magnitude smaller than the experimental result of 0.131 S/m.^30^ Nevertheless, we can still compare the ratios of different contributions in eq 4. For the equimolar LiTFSI electrolyte, the ratio of “distinct” to “self” term for cations is σ_++_/σ_+_ = −0.61. The corresponding value for anions, σ_––_/σ_–_, is −0.68. The experimental values are −0.66 and −0.80, respectively.^30^ Our simulations reproduce the effect of anticorrelations in motions of ions of the same charge, reducing the overall contribution to the conductivity by about 2/3. The ratio of cation–anion anticorrelations to the total conductivity, σ_+‑_/σ is −0.18, in good agreement with the experimental result of −0.24.^30^ We can therefore conclude that our simulations correctly reproduce the effects of ion correlations, and the underestimated conductivities are the consequence of an overestimated viscosity of the solvent.

For the practical application of the ion-conducting electrolyte, not only its conductivity is an important parameter but also the cation transport number, *t*_+_. A commonly used estimate is based on the diffusion coefficients of ions (experimentally accessible from pulsed-field gradient nuclear magnetic resonance measurements)

5 $$t_{+}^{D}=\frac{D_{+}}{D_{+}+D_{-}}$$

However, *t*_+_^*D*^ can be a meaningful characteristic of ion transport only when the ion–ion correlations are negligible. Otherwise, the transport number should be calculated from the basic definition as the ratio of the current transported by cations to the total current transported by all ions. Using the notation from eq 4, one obtains

6 $$t_{+}^{c}=\frac{\sigma_{+}+\sigma_{++}-\sigma_{+-}}{\sigma }$$

When σ_++_ = σ_––_ = σ_+–_ = 0, *t*_+_^*c*^ reduces to *t*_+_^*D*^. During the stationary charging/discharging of a battery, only cations are transported in the electrolyte between the electrodes. The transport number under anion blocking conditions can be defined as^30,52^

7 $$t_{+}^{\mathrm{abc}}=\frac{\sigma_{+}+\sigma_{++}-\frac{{\left(\sigma_{+-}\right)}^{2}}{\sigma_{-}+\sigma_{--}}}{\sigma }$$

Note that in refs 26, 30, and 52, *t*_+_^*D*^ are named Li^+^ transport numbers, whereas *t*_+_^*c*^ and *t*_+_^*abc*^ are called transference numbers. For consistency, we will use the same terminology from now on.

The values of the Li^+^ transport and transference numbers, estimated from our MD simulations, are listed in Table 2. The diffusion coefficient-based transport numbers increase with salt concentration, approaching the value 0.5 at the highest salt concentration as a consequence of *D*_+_ and *D*_–_ becoming roughly equal. The *t*_+_^*D*^ = 0.48 calculated for the equimolar LiTFSI/G4 electrolyte is only slightly lower than the experimental value of 0.51.^30^ In the least concentrated 1:20 LiTFSI electrolyte, the transference number *t*_+_^*c*^, including correlations, is smaller than *t*_+_^*D*^ due to destructive contribution from σ_+‑_. In other systems, *t*_+_^*c*^ > *t*_+_^*D*^, as a result of cation–anion anticorrelated movements and of the −2σ_+‑_ term becoming positive (Cf. Figure 10). In the most concentrated LiTFSI electrolyte, *t*_+_^*c*^ = 0.54, in a quite good agreement with the experimental result of 0.58.^30^

With increasing salt content, the cation transference numbers under anion blocking conditions decrease. Although the value of 0.23 obtained for the most concentrated LiTFSI solution is significantly higher than the experimental value of 0.025,^30^ it is evident that *t*_+_^*abc*^ are significantly lower than *t*_+_^*c*^ or *t*_+_^*D*^, in agreement with experimental knowledge. At the 1:8 and 1:5 Li/O_g_ ratios, *t*_+_^*abc*^ are practically equal for both salts. Regardless of the anion, ion–ion correlations, increasing the conductivity of solvate ionic liquids, simultaneously reduce the *t*_+_^*abc*^ transference numbers to low values, implying limited performance under real operation conditions of a battery. In this respect, LiFPFSI does not seem to be any better than LiTFSI. It should be noted that the transference numbers are strongly affected by the Li^+^ interactions with solvent molecules, modifying the σ_+‑_ contribution. In particular, the formation of long-living [Li­(G4)]^+^ solvates, turning the system into a solvate IL, increases the cation–anion correlations, reducing the *t*_+_^*abc*^ values.

## 4 Conclusions

We used MD simulations to compare Li-conducting electrolytes based on tetraglyme solvent and two salts with isomeric anions, LiTFSI and LiFPFSI. The electrolytes were characterized by the parameters relevant to their structure (RDFs, CNs, and composition of the solvation shells) and transport properties (Li–O ACFs, diffusion coefficients, and conductivity).

The structural data agree with the predictions based on quantum chemical calculations and AIMD simulations for small systems,^36^ indicating slightly weaker Li^+^ binding to FPFSI anions and resulting in a small increase in the amount of Li-solvent complexes and more cations in monodentate coordination in LiFPFSI solutions in G4. At the highest salt content, only a small fraction of solvent molecules remain noncoordinating to cations. Both coordination numbers and autocorrelation functions for Li–O interactions confirm that very stable [Li­(G4)]^+^ solvates are formed in the electrolytes, regardless of the salt type, and the equimolar salt/G4 solutions behave as solvate ionic liquids.

The latter findings are corroborated by the analysis of diffusion coefficients and correlations in ion–ion motions. Consistently with the picture of solvate ILs, the cation–anion anticorrelated movements become more pronounced as the salt concentration approaches the equimolar salt/solvent ratio, accordingly increasing the positive contribution to the total conductivity. The effect is stronger for LiFPFSI; as a result, the conductivity of LiFPFSI electrolytes is appreciably higher than that of LiTFSI solutions. This finding agrees with the experimental data of ref 33, showing that at *T* = 303 K and the 1:8 Li/O ratio, the conductivity of the LiFPFSI/PEO electrolyte is higher than for the LiTFSI-based system. However, the practical applicability of tetraglyme solutions of both salts appears to be constrained by the low Li^+^ transference numbers under the anion blocking conditions. More broadly, our present results underscore the critical importance of analyzing ion–ion correlations in MD studies of concentrated salt solutions, as these correlations strongly influence cation transport and electrolyte performance.

## Supplementary Material



## Notes

Details of force field development, X-ray static structure factors from different variants of the FF, changes of CNs during the MD simulations, average values of CNs, comparison of CNs for LiTFSI and LiFPFSI, Li–F RDFs, Venn diagrams for LiTFSI/G4 electrolytes, changes in the distributions of dihedral angles at different electrolyte concentrations, autocorrelation functions for Li–O interactions, parameters of fits to the Li–O ACFs, sample plots of MSD vs time, flowchart of force field parametrization, and final force field parameters (PDF)
