# Supplementary material for: Molecular Dynamics Study on Tetraglyme Solutions of Two Lithium Salts with Isomeric Anions: LiTFSI and LiFPFSI
Source: J Phys Chem B. 2026 Jan 30;130(6):1902–14. doi: 10.1021/acs.jpcb.5c07631 (PMC13298901; doi:10.1021/acs.jpcb.5c07631)
Supplement: Supplementary file 1 [file jp5c07631_si_001.pdf]

# Molecular Dynamics Study on Tetraglyme Solutions of Two Lithium Salts with Isomeric Anions: LiTFSI and LiFPFSI

Piotr Kubisiak,[a] Chiara Nicotri,[a,b] Andrzej Eilmes[a,*]

[a] *Faculty of Chemistry, Jagiellonian University, Gronostajowa 2, 30-387 Kraków, Poland*

[b] *Department of Applied Science and Technology, Politecnico di Torino,*
*Corso Duca degli Abruzzi 24, 10129 Torino, Italy*

[*] *e-mail: eilmes@chemia.uj.edu.pl*

**Supporting Information**

Table S1. Interatomic distances and $Li^+$ binding energies in Li-anion complexes obtained from the calculations using MP2/aug-cc-pVDZ method and two variants of the force field. FF1/FF1-sc variant uses the NBFIX options.

| system/ parameter | MP2 | FF0 | FF1/FF1-sc |
|---|---|---|---|
| **Li-TFSI (i)** | | | |
| $E_b$, kcal/mol | -136.06 | -135.68 | -135.69 |
| d(Li-$O_{an}$), Å | 1.84 | 1.85 | 1.85 |
| d(Li-N), Å | 3.37 | 3.19 | 3.24 |
| d(Li-F), Å | 3.20 | 3.17 | 3.18 |
| **Li-TFSI (ii)** | | | |
| $E_b$, kcal/mol | -136.10 | -139.14 | -139.11 |
| d(Li-$O_{an}$), Å | 1.84 | 1.85 | 1.85 |
| d(Li-N), Å | 3.25 | 3.04 | 3.09 |
| d(Li-F), Å | 3.68, 4.09 | 3.71, 4.01 | 3.70, 4.01 |
| **Li-FPFSI (i)** | | | |
| $E_b$, kcal/mol | -134.47 | -132.44 | -127.93 |
| d(Li-$O_{an}$), Å | 1.91, 2.02 | 1.88, 2.05 | 1.87, 1.93 |
| d(Li-N), Å | 3.24 | 2.93 | 3.06 |
| d(Li-F), Å | 1.98 | 1.60 | 1.96 |
| **Li-FPFSI (ii)** | | | |
| $E_b$, kcal/mol | -132.51 | -133.55 | -133.52 |
| d(Li-$O_{an}$), Å | 1.85, 1.85 | 1.85, 1.86 | 1.85, 1.86 |
| d(Li-N), Å | 3.33 | 3.10 | 3.10 |
| d(Li-F), Å | 3.31 | 3.22 | 3.22 |

Table S2. Li-O distances and $Li^+$ binding energies in the $[Li(G4)]^+$ complex obtained from the MP2/aug-cc-pVDZ calculations and different variants of the force field.

| | MP2 | FF0 | FF0c | FF1 | FF1-sc |
|---|---|---|---|---|---|
| scaling factors: | | | | | |
| charges | - | 1.0 | 0.7 | 1.0 | 0.9 |
| polarizabilities | - | 1.0 | 1.0 | 0.7 | 0.7 |
| $E_b$, kcal/mol | -118.7 | -141.1 | -124.3 | -126.6 | -119.1 |
| d(Li-$O_g$), Å | 1.97 | 1.78 | 1.79 | 1.90 | 1.90 |
| | 1.98 | 1.79 | 1.79 | 1.91 | 1.91 |
| | 2.02 | 2.05 | 2.08 | 1.97 | 1.97 |
| | 2.08 | 2.12 | 2.12 | 2.04 | 2.04 |
| | 2.08 | 2.15 | 2.15 | 2.04 | 2.05 |

Table S3. Positions of the maxima in the Li-O RDFs, coordination numbers and the densities for the 1:8 LiTFSI/LiFPFSI solutions in G4 obtained from the NpT MD simulations at T = 303 K and p = 1 atm.

| | FF1-sc | | FF1 | |
|---|---|---|---|---|
| | $r_m$, Å | CN | $r_m$, Å | CN |
| **LiTFSI** | | | | |
| Li-$O_{an}$ | 1.97 | 1.71 | 1.96 | 1.04 |
| Li-$O_g$ | 1.91 | 2.62 | 1.91 | 3.43 |
| **LiFPFSI** | | | | |
| Li-$O_{an}$ | 1.96 | 1.48 | 1.97 | 0.96 |
| Li-$O_g$ | 1.91 | 2.87 | 1.91 | 3.52 |

| | density, g/cm$^3$ | | |
|---|---|---|---|
| | FF1-sc | FF1 | exp. |
| LiTFSI | 1.212 | 1.242 | 1.316 [a] |
| LiFPFSI | 1.228 | 1.247 | - |

[a] from *J. Phys. Chem. C* **2011**, *115*, 18384-18394.

Table S4. Average values of the CNs calculated from the MD simulations at the 3 Å Li-O distance.

| Li:$O_g$ ratio | TFSI | | | FPFSI | | | | |
|---|---|---|---|---|---|---|---|---|
| | $O_g$ | $O_{an}$ | total[a] | $O_g$ | $O_{SO}$ | $O_{SF}$ | $O_{an}$[b] | total |
| 1:20 | 4.31 | 0.30 | 4.61 | 4.30 | 0.16 | 0.11 | 0.27 | 4.57 |
| 1:8 | 3.99 | 0.63 | 4.62 | 3.99 | 0.39 | 0.25 | 0.64 | 4.63 |
| 1:5 | 3.51 | 1.03 | 4.54 | 3.56 | 0.55 | 0.44 | 0.99 | 4.55 |

[a] CN(total) = CN($O_g$) + CN($O_{an}$)
[b] for FPFSI CN($O_{an}$) = CN($O_{SO}$) + CN($O_{SF}$)

Table S5. Parameters of the fit of exp[-($t$/$\tau$)$^{\alpha}$] function to the autocorrelation functions of Li-O residence times. $\tau$ values are in ns.

| Li:$O_g$ ratio | TFSI | | | | FPFSI | | | | | |
|---|---|---|---|---|---|---|---|---|---|---|
| | Li-$O_{an}$ | | Li-anion | | Li-$O_{SO}$ | | Li-$O_{SF}$ | | Li-anion | |
| | $\tau$ | $\alpha$ | $\tau$ | $\alpha$ | $\tau$ | $\alpha$ | $\tau$ | $\alpha$ | $\tau$ | $\alpha$ |
| 1:20 | 6.0 | 0.67 | 18.8 | 0.85 | 9.4 | 0.76 | 5.1 | 0.68 | 18.6 | 0.72 |
| 1:8 | 10.1 | 0.55 | 40.9 | 0.80 | 17.2 | 0.71 | 9.1 | 0.62 | 43.7 | 0.82 |
| 1:5 | 15.8 | 0.46 | 85.7 | 0.74 | 24.6 | 0.57 | 11.8 | 0.49 | 84.0 | 0.74 |

| Li:$O_g$ ratio | TFSI | | | | FPFSI | | | |
|---|---|---|---|---|---|---|---|---|
| | Li-$O_g$ | | Li-G4 | | Li-$O_g$ | | Li-G4 | |
| | $\tau$ | $\alpha$ | $\tau$ | $\alpha$ | $\tau$ | $\alpha$ | $\tau$ | $\alpha$ |
| 1:20 | 131 | 0.41 | 230 | 0.93 | 121 | 0.37 | 255 | 0.96 |
| 1:8 | 474 | 0.36 | 502 | 0.91 | 776 | 0.33 | 629 | 0.91 |
| 1:5 | 1162 | 0.41 | 1021 | 0.85 | 2173 | 0.35 | 1109 | 0.89 |

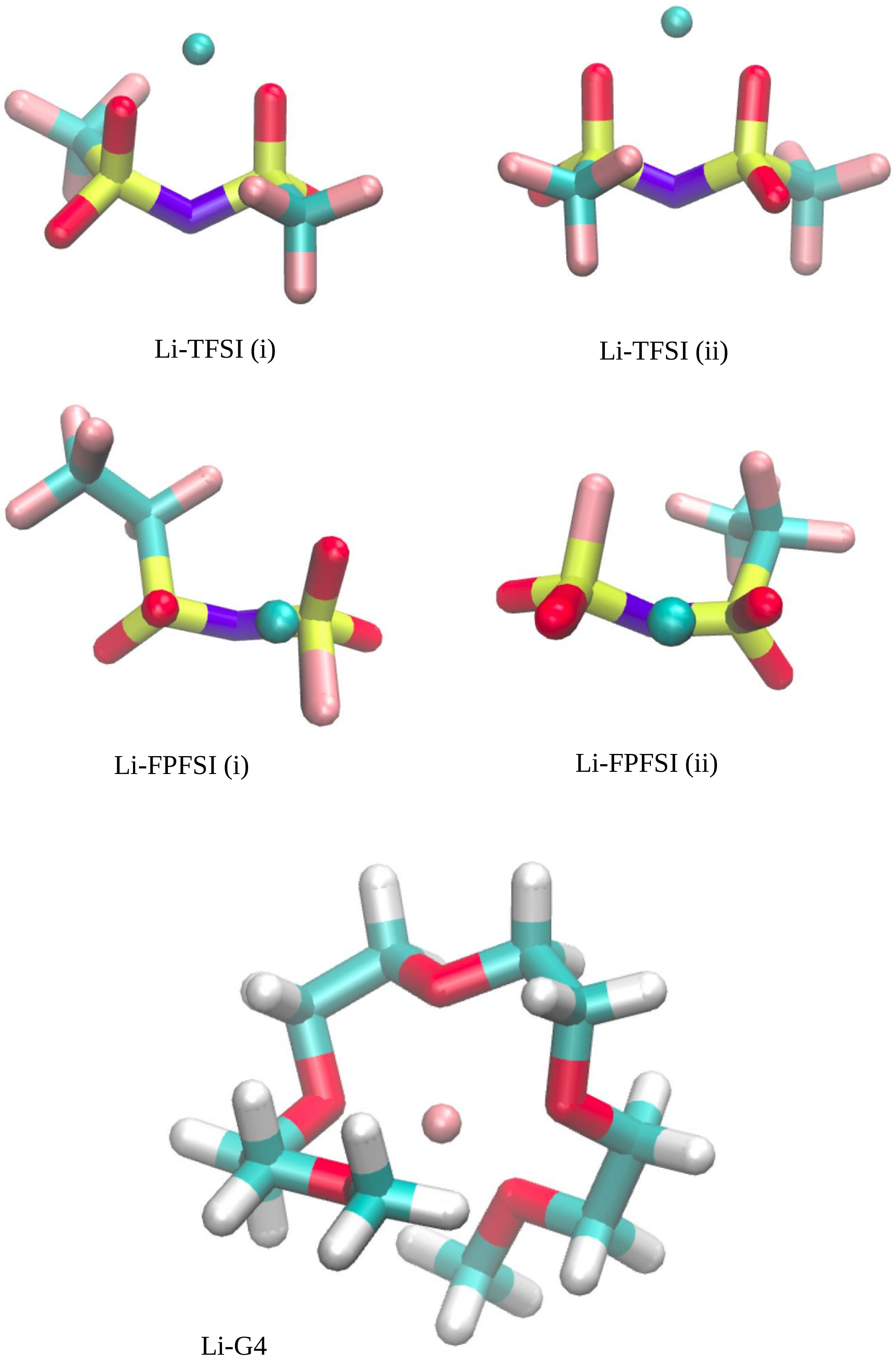


Figure S1. Structures of the Li-TFSI, Li-FPFSI and $[Li(G4)]^+$ complexes used in the FF development.

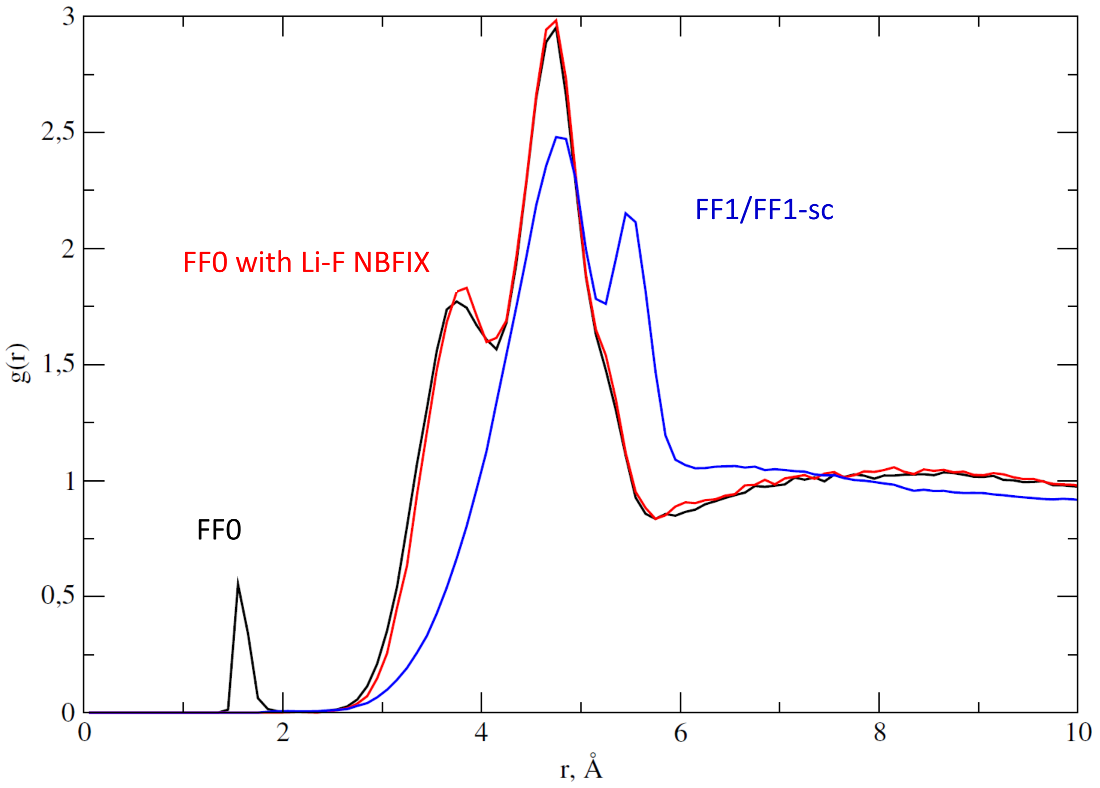


Figure S2. Li-F RDFs for the LiTFSI 1:8 electrolyte simulated in different variants of the FF.

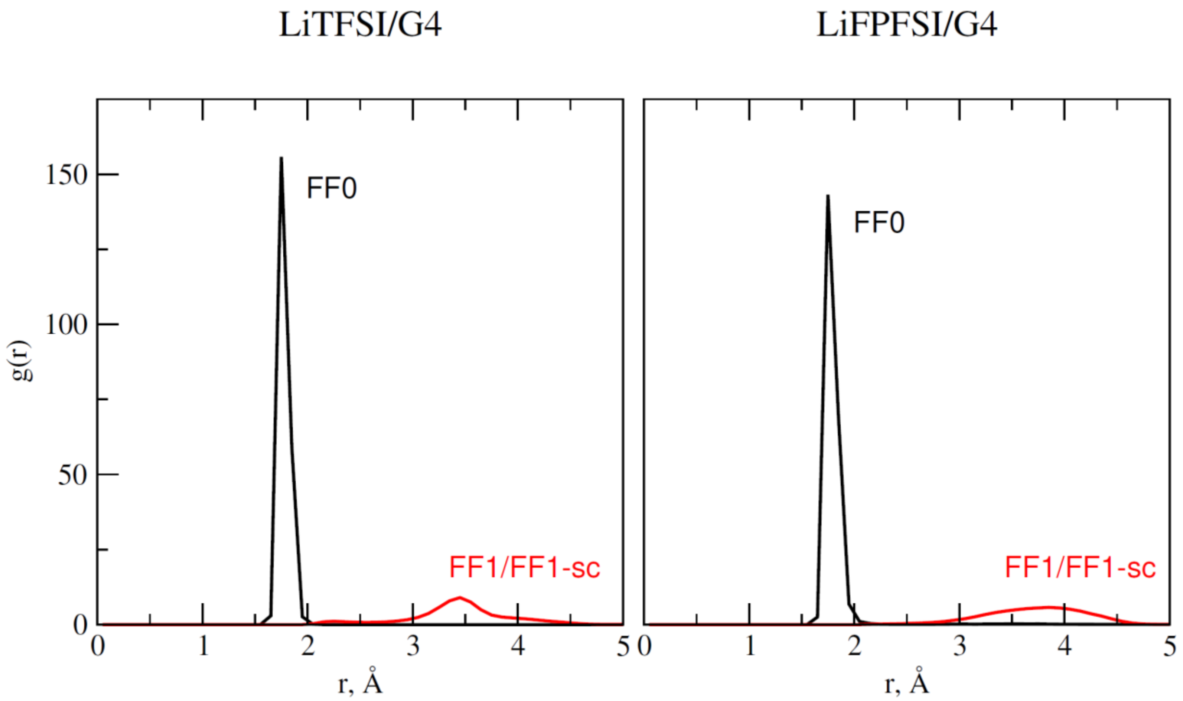


Figure S3. Li-N RDFs in the LiTFSI and LiFPFSI 1:8 electrolytes simulated in different variants of the FF.

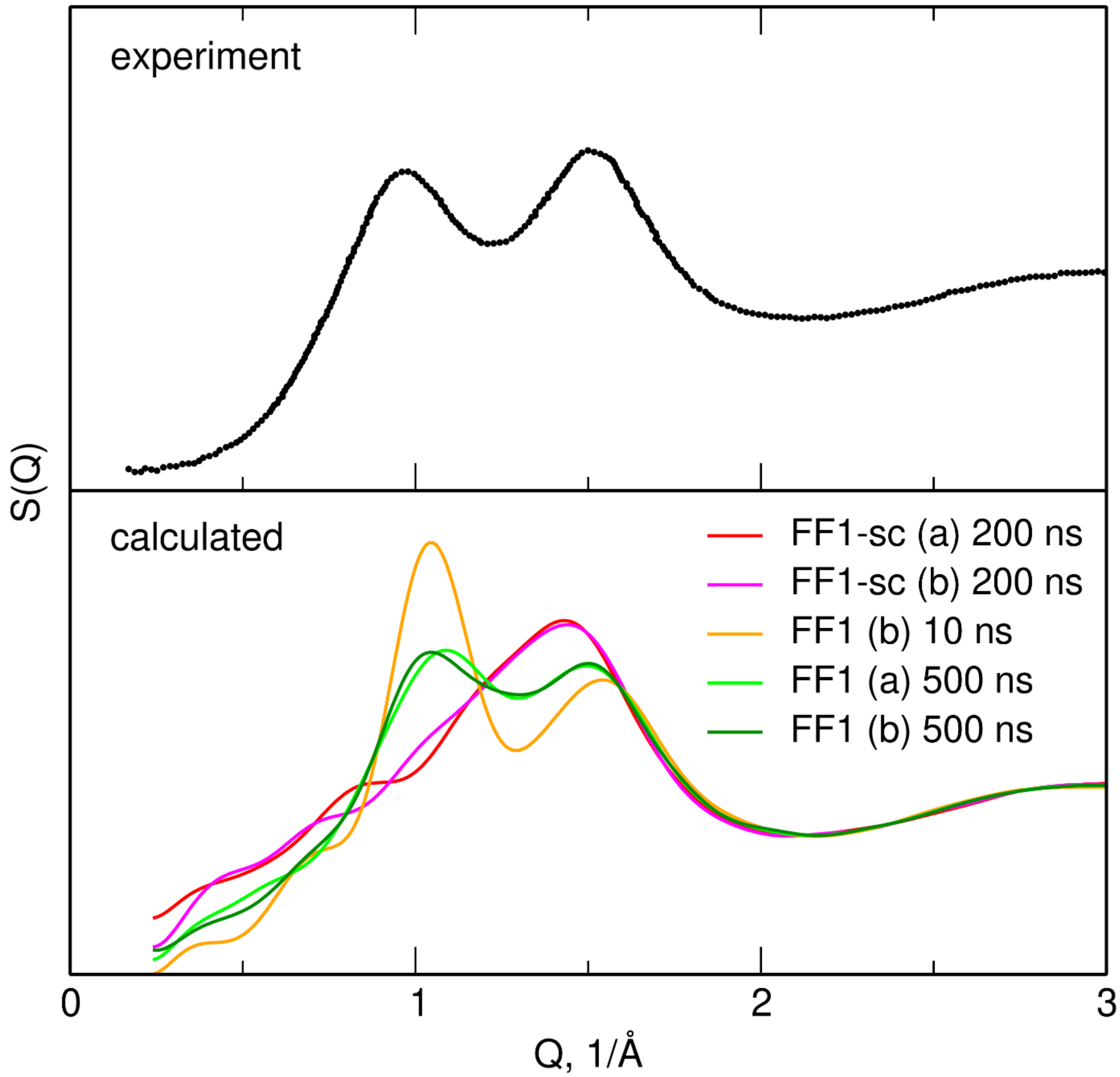


Figure S4. X-ray static structure factors calculated for the 1:5 LiTFSI electrolytes of type *a* and *b* at different stages of the MD simulations. Experimental data from *Phys. Chem. Chem. Phys.* **2015**, *17*, 8248-8257.

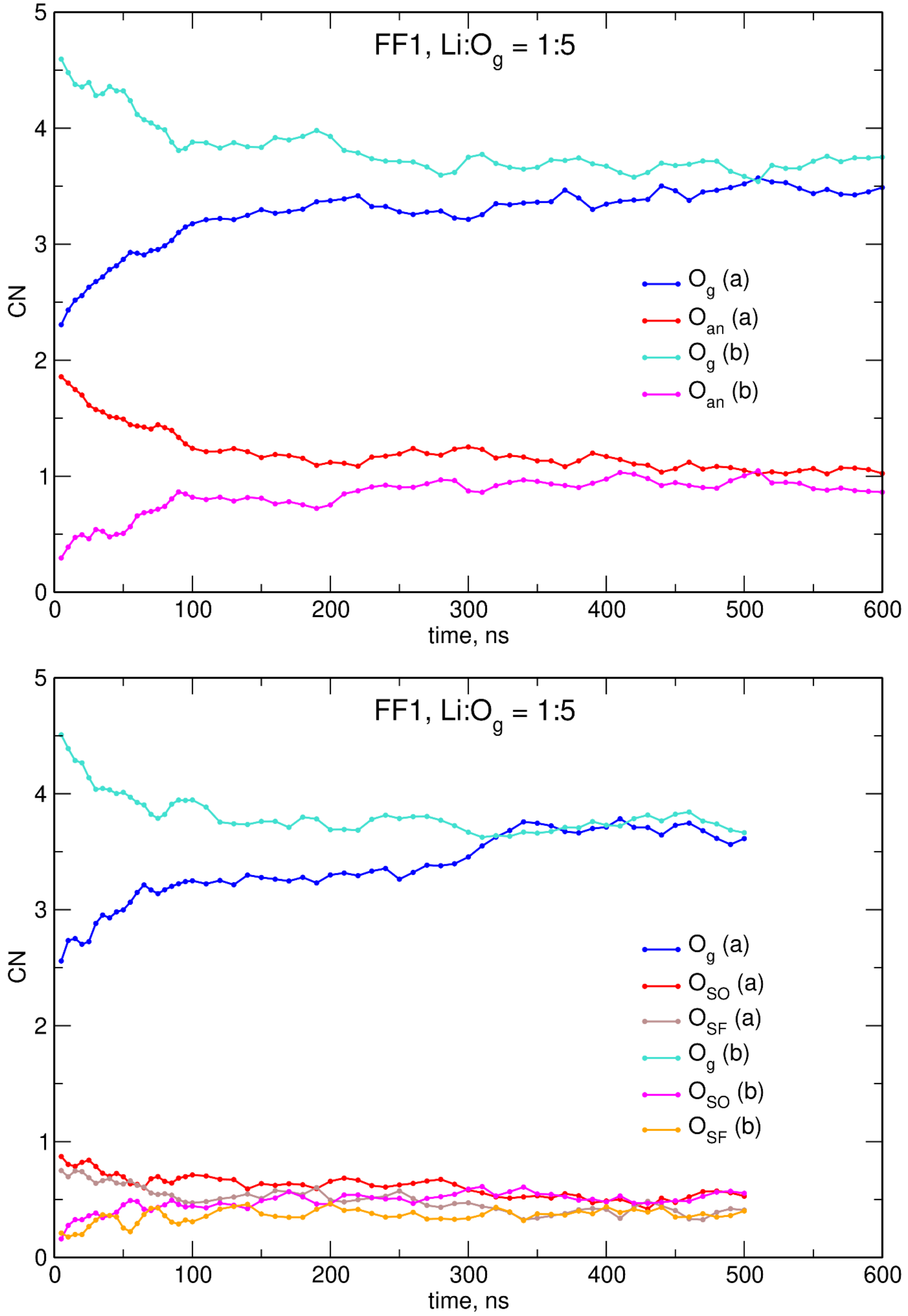


Figure S5. Evolution of the average Li-O CNs during the MD simulations for the LiTFSI (top panel) and LiFPFSI (bottom panel) 1:5 electrolytes of types *a* and *b*.

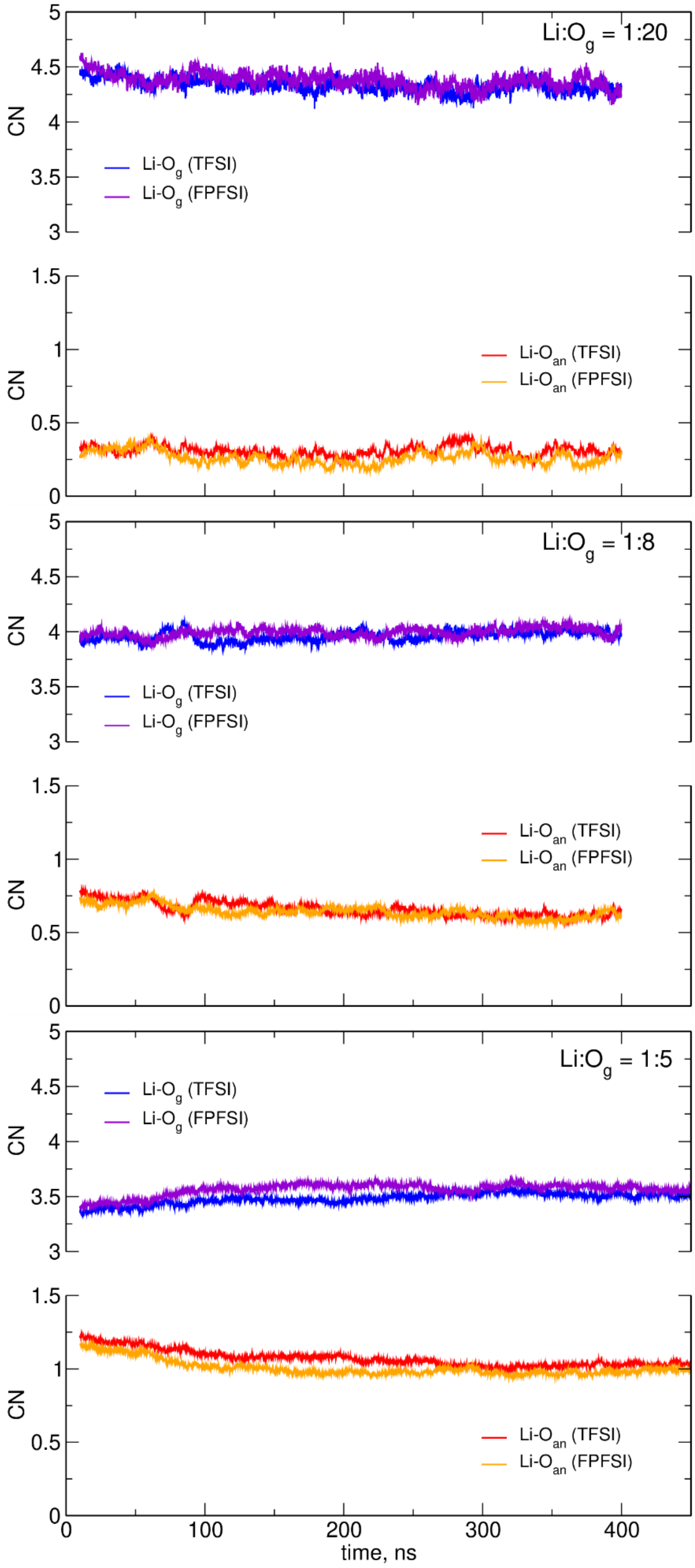


Figure S6. Changes of average CNs in MD simulations for LiTFSI and LiFPFSI electrolytes.

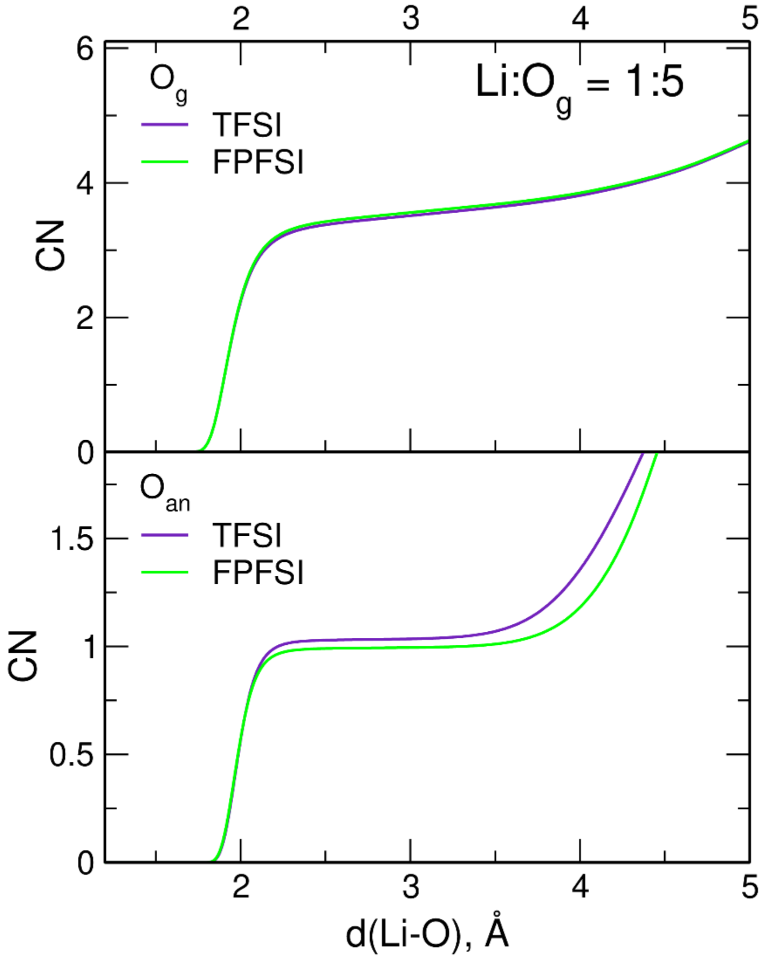


Figure S7. Comparison of the running CNs for Li-O pairs in the 1:5 LiTFSI and LiFPFSI electrolytes.

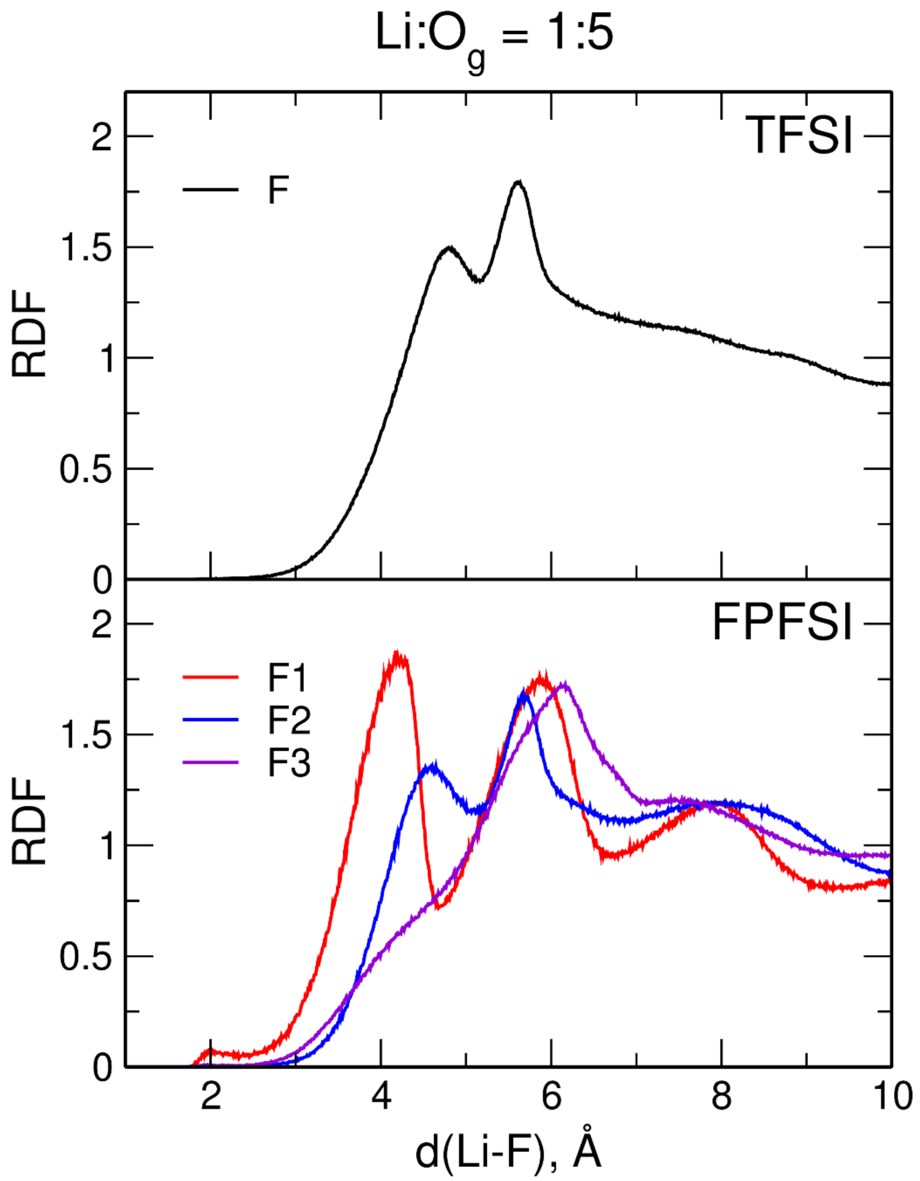


Figure S8. Li-F RDFs for the 1:5 LiTFSI and LiFPFSI electrolytes.

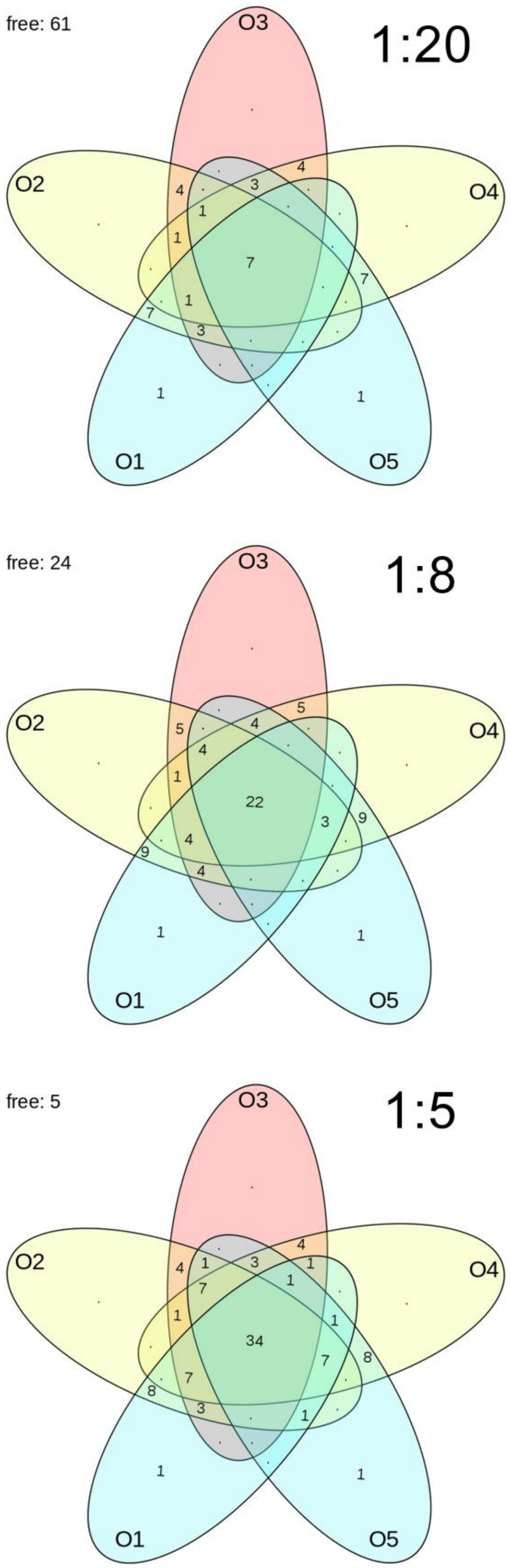


Figure S9. Venn diagrams showing the connectivity between the $Li^+$ ions and O atoms of tetraglyme in LiTFSI electrolytes. Values smaller than 1% are displayed as dots.

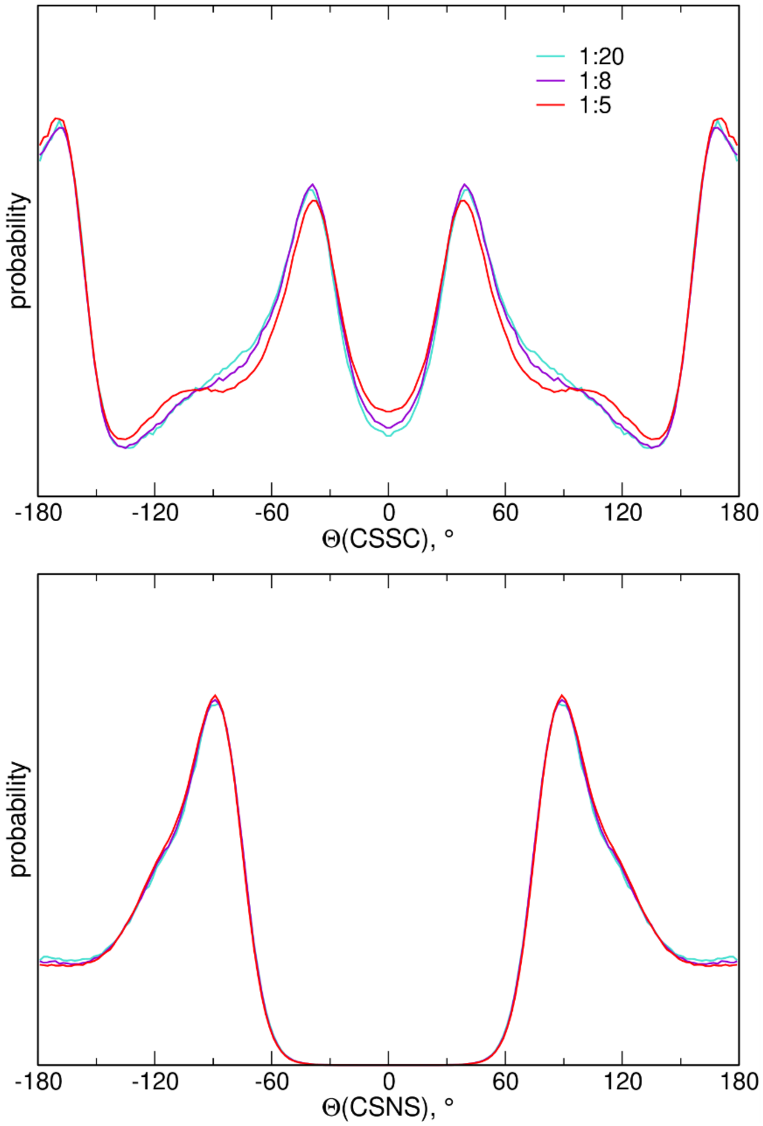


Figure S10. Distributions of the values of dihedral angles in TFSI anions in LiTFSI/G4 electrolytes at different Li:$O_g$ ratios.

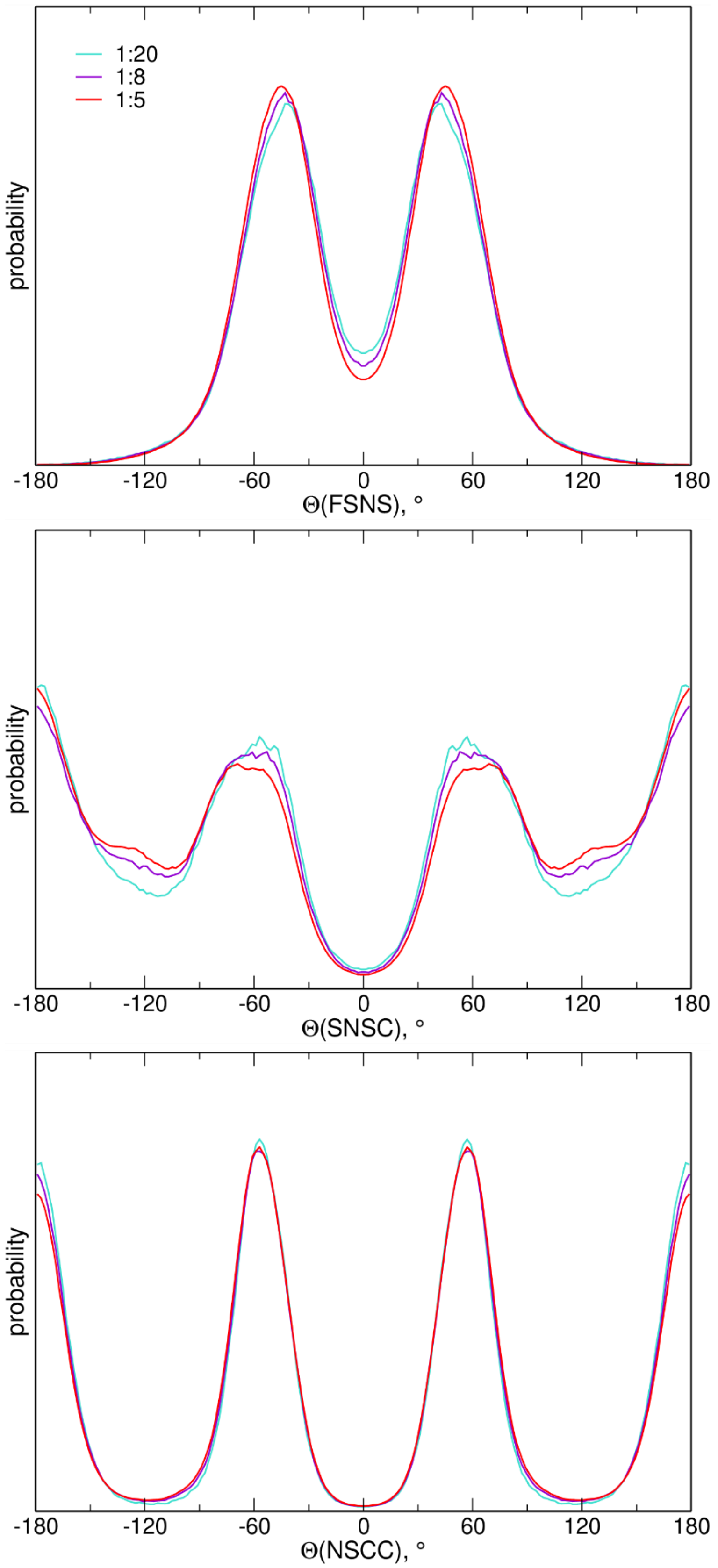


Figure S11. Distributions of the values of dihedral angles in FPFSI anions in LiFPFSI/G4 electrolytes at different Li:$O_g$ ratios.

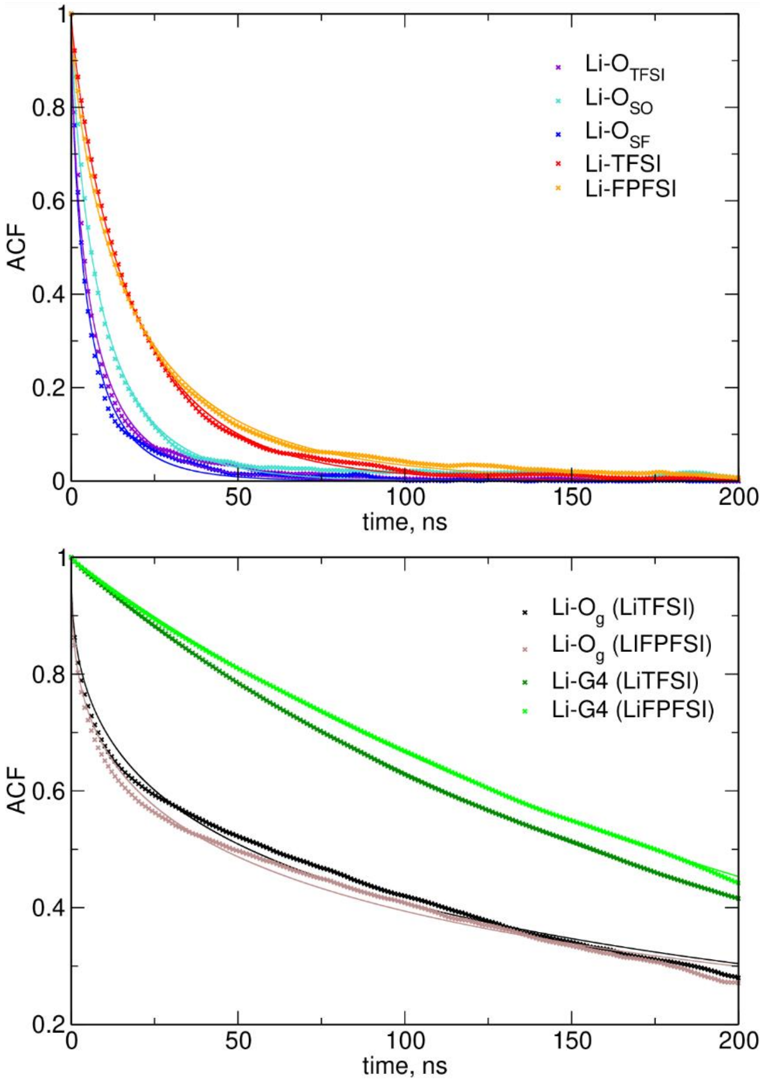


Figure S12. Autocorrelation functions for $Li^+$ interactions with O atoms, anions or solvent molecules in the Li:O$_g$ = 1:20 LiTFSI and LiFPFSI electrolytes. Lines are fits to the data.

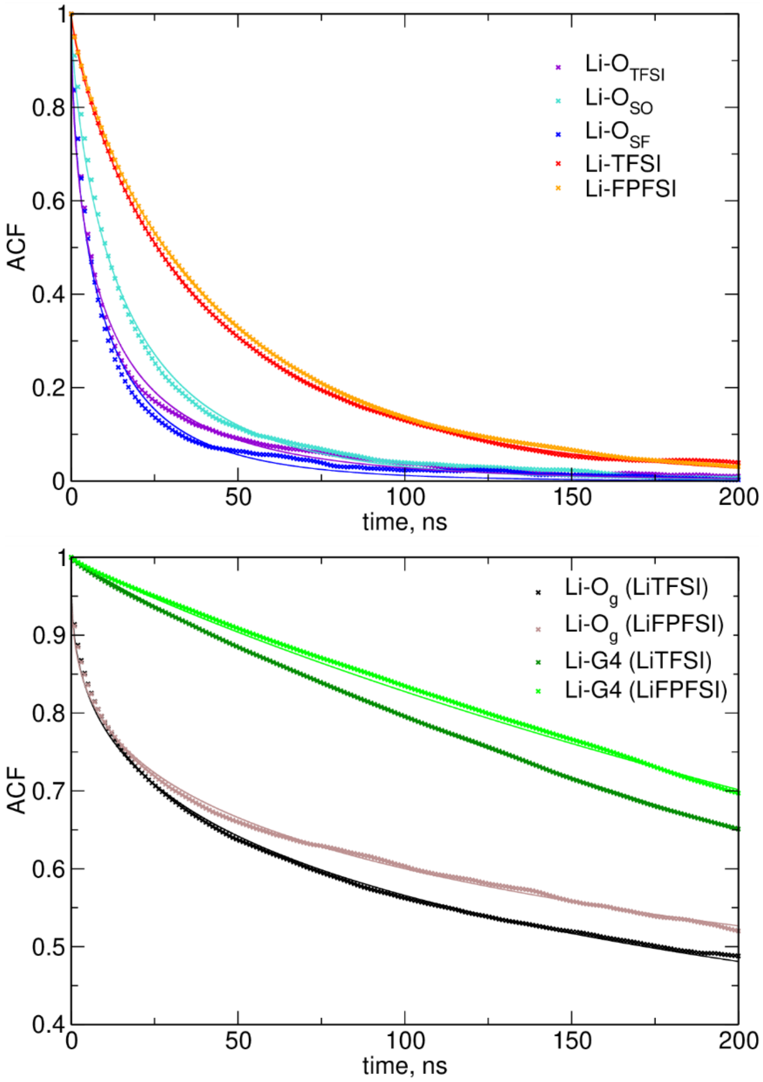


Figure S13. Autocorrelation functions for $Li^+$ interactions with O atoms, anions or solvent molecules in the Li:$O_g$ = 1:8 LiTFSI and LiFPFSI electrolytes. Lines are fits to the data.

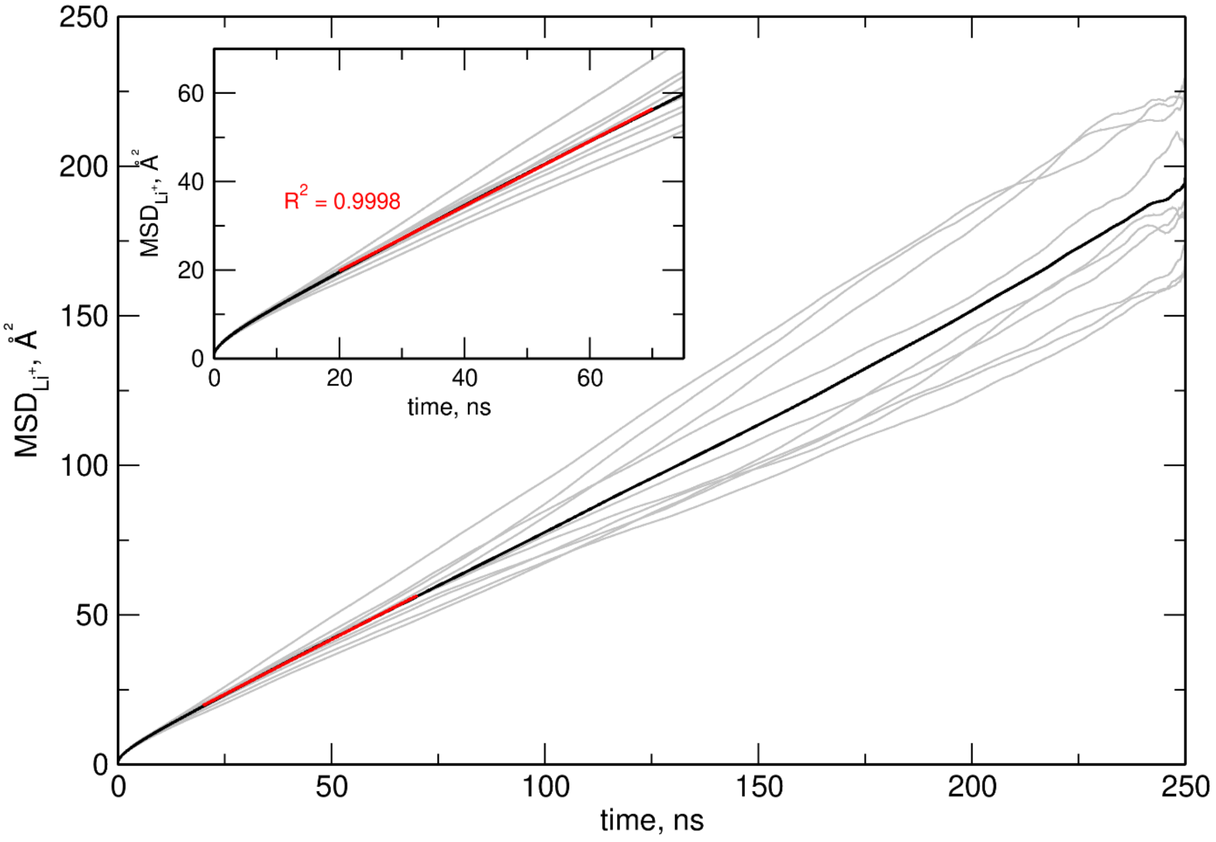


Figure S14. Mean square displacements of $Li^+$ ions in the 1:5 LiTFSI electrolyte. Gray lines are the data for individual trajectories, black line is the average over the trajectories and the red line is the linear fit to the data used to estimate the diffusion coefficient $D_{Li}$.

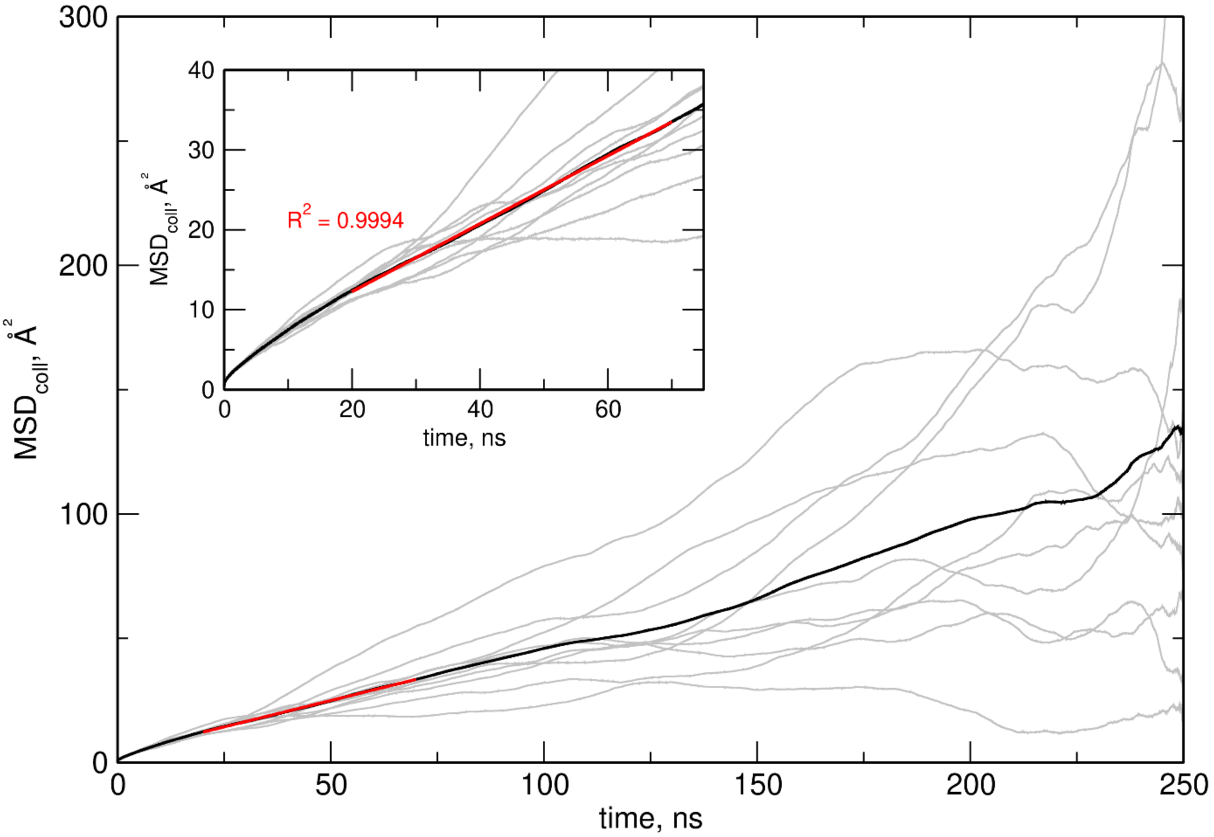


Figure S15. Collective mean square displacements of ions in the 1:5 LiTFSI electrolyte. Gray lines are the data for individual trajectories, black line is the average over the trajectories and the red line is the linear fit to the data used to estimate the conductivity of the system.

## Appendix A – force field parameterization

Table S6. Sources of parameters for the initial force field FF0.

| | TFSI anion | FPFSI anion | G4 molecule | $Li^+$ |
|---|---|---|---|---|
| bonded params. | fit using FF Toolkit (ref. S1) | | | - |
| L-J potential | ref. S2 | | ref. S3 | ref. S4 |
| charges | fit to electrostatic potential | | | +1 |
| polarizabilities | APPLE&P (ref. S5) | | ref. S6 | 0 |

(S1) Mayne, C. G.; Saam, J.; Schulten, K.; Tajkhorshid, E.; Gumbart, J. C. Rapid Parameterization od Small Molecules Using the Force Field Toolkit. *J. Comp. Chem.* **2013**, *34*, 2757-2770.

(S2) Köddermann, T.; Paschek, D.; Ludwig, R. Molecular Dynamics Simulations of Ionic Liquids. A Reliable Description of Structure, Thermodynamics and Dynamics. *ChemPhysChem* **2007**, *8*, 2464-2470.

(S3) Anderson, P. M.; Wilson, M. R. Developing a Force Field for Simulation of Poly(ethylene oxide) Based upon Ab Initio Calculations of 1,2-Dimethoxyethane. *Mol. Phys.* **2005**, *103*, 89-97.

(S4) Jensen, K. P.; Jorgensen, W. L. Halide, Ammonium, and Alkali Metal Ion Parameters for Modeling Aqueous Solutions. *J. Chem. Theory Comput.* **2006**, *2*, 1499-1509.

(S5) Borodin, O. Polarizable Force Field Development and Molecular Dynamics Simulations of Ionic Liquids. *J. Phys. Chem. B* **2009**, *113*, 11463-11478.

(S6) Borodin, O.; Smith, G. D. Development of Quantum Chemistry-Based Force Fields for Poly(ethylene oxide) with Many-Body Polarization Interactions. *J. Phys. Chem. B* **2003**, *107*, 6801-6812.

Scheme S1. A flowchart presenting the successive improvement of the initial force field toward the final force field (FF1).

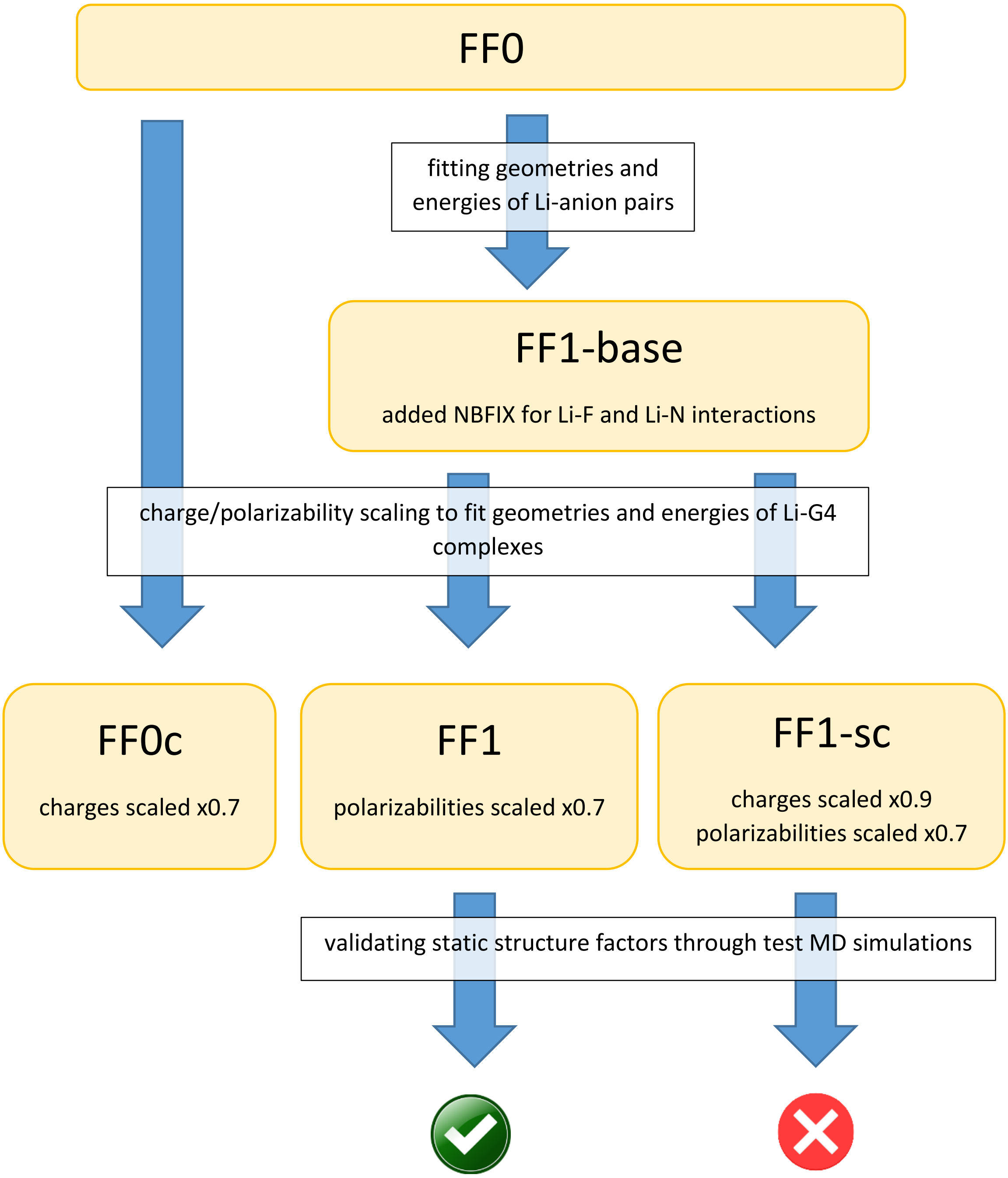

Parameters of the final force field (FF1)

Atom types:

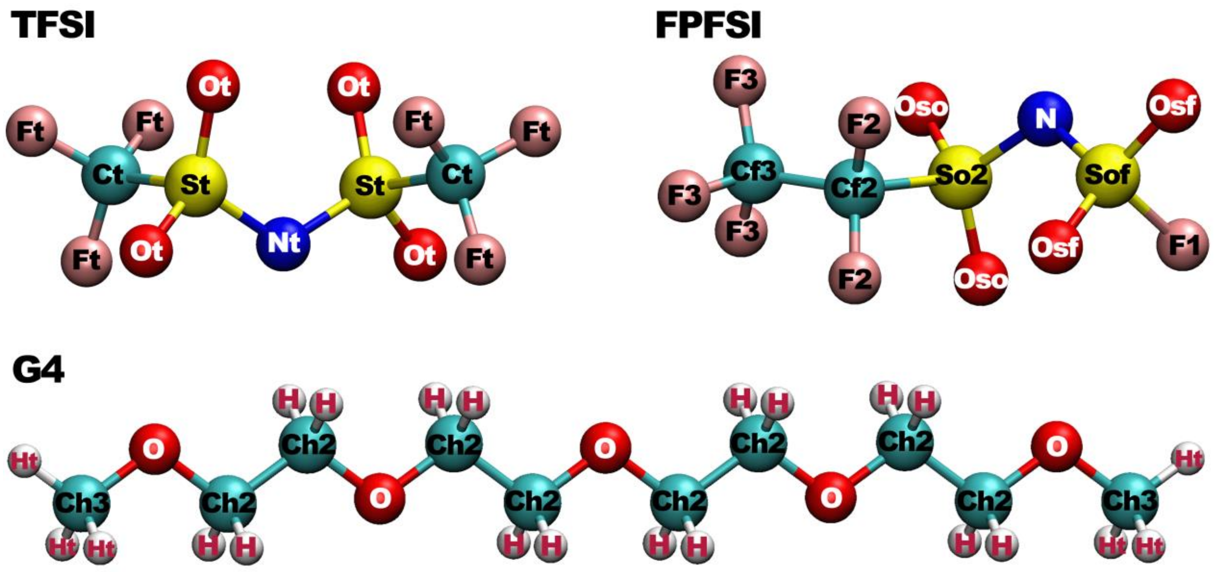


FF parameters in the NAMD format (.prm file):

```
BONDS
!!!!!!!!!!!!!!!!!!!!!!!!!!!!!!!!!!!!!
!  ********* V(bond) = Kb(b - b0)**2
!
!Kb: kcal/mole/A**2
!b0: A
!
!atom type Kb    b0
!!!!!!!!!!!!!!!!!!!!!!!!!!!!!!!!!!!!!
!! G4
Ch2    O      334.1160   1.4057
Ch2    H      335.3486   1.0979
O      Ch3    343.3620   1.4120
Ch2    Ch2    311.5529   1.4962
Ht     Ch3    347.3186   1.0941
DCh     Ch2     500.0            0.000
DCh     Ch3     500.0            0.000
DO      O       500.0            0.000


!! TFSI
Ct     Ft     369.4140   1.3467
Nt     St     287.5512   1.6430
St     Ct     154.1592   1.8162
St     Ot     576.3862   1.4860
DNt    Nt      500.0      0.000
DSt    St      500.0      0.000
DOt     Ot       500.0     0.000
```

```
DCt    Ct      500.0    0.000
DFt    Ft      500.0    0.000


!! FPFSI
Sof   Osf  594.3674   1.4793
Cf3   F3   379.6684   1.3457
So2   Oso  579.5195   1.4856
N     Sof  297.9737   1.6466
N     So2  287.3220   1.6538
Sof   F1   200.9001   1.6811
Cf3   Cf2  241.7714   1.5155
Cf2   So2  139.9310   1.8605
Cf2   F2   366.1008   1.3524
DCf   Cf2   500.0     0.000
DCf   Cf3   500.0     0.000
DOs   Oso   500.0     0.000
DOs   Osf   500.0     0.000
DSo   So2   500.0     0.000
DSo   Sof   500.0     0.000
DN    N     500.0     0.000
DF1   F1    500.0     0.000
DF2   F2    500.0     0.000
DF3   F3    500.0     0.000


ANGLES
!!!!!!!!!!!!!!!!!!!!!!!!!!!!!!!!!!!!!!!!!!!!!!!!
! ********* V(angle) = Ktheta(Theta - Theta0)**2
!
!
!Ktheta: kcal/mole/rad**2
!Theta0: degrees
!
!atom types     Ktheta    Theta0
!!!!!!!!!!!!!!!!!!!!!!!!!!!!!!!!!!!!!!!!!!!!!!!!!

!! G4
O     Ch2  H     49.6078    110.8074
Ch2   O    Ch2   52.6717    108.9879
H     Ch2  H     41.9504    109.1480
Ch2   Ch2  O     68.4643    105.0460
O     Ch3  Ht    20.7423    106.9560
Ch2   Ch2  H     39.5035    110.8623
Ch2   O    Ch3   60.7466    107.3984
Ht    Ch3  Ht    56.7389    109.4237

!!TFSI
St    Ct   Ft    12.7827    106.5411
St    Nt   St    47.1384    114.6326
Ct    St   Nt    61.7910    100.5282
Ot    St   Nt    43.7687    118.0519
Ot    St   Ot    82.6031    125.3952
Ct    St   Ot    74.3614    103.6325
Ft    Ct   Ft    97.7929    109.4619
```

```
!! FPFSI
Osf    Sof   Osf   95.3106    121.1473
Cf2    So2   Oso   56.1315    100.7354
F2     Cf2   F2   116.9483    109.8510
Cf3    Cf2   So2   44.9009    117.4049
Oso    So2   N     40.3585    111.5267
Cf2    So2   N     84.0786    102.7379
N      Sof   F1    85.5785    100.9115
F3     Cf3   F3    90.8676    110.7452
Osf    Sof   F1   102.3451    103.0045
F3     Cf3   Cf2   26.9316    109.3035
So2    N     Sof   45.2924    116.0282
Oso    So2   Oso   86.1937    122.4139
N      Sof   Osf   26.0247    112.7772
F2     Cf2   So2   6.9829     107.5355
Cf3    Cf2   F2    56.8964    106.8862


DIHEDRALS
!!!!!!!!!!!!!!!!!!!!!!!!!!!!!!!!!!!!!!!!!!!!!!!!!!!!!
! ******** V(dihedral) = Kchi(1 + cos(n(chi) - delta))
!
!Kchi: kcal/mole
!n: multiplicity
!delta: degrees
!
!atom types             Kchi    n   delta
!!!!!!!!!!!!!!!!!!!!!!!!!!!!!!!!!!!!!!!!!!!!!!!!!!!!!

!! G4
Ch2    Ch2   O     Ch2   0.113          3     0.00
Ch2    Ch2   O     Ch2   0.648          2     0.00
Ch2    Ch2   O     Ch3   0.106          3     0.00
Ch2    Ch2   O     Ch3   0.274          2     0.00
Ch2    O     Ch3   Ht    0.348          3     0.00
H      Ch2   Ch2   H     0.072          3     0.00
H      Ch2   O     Ch2   0.401          3     0.00
H      Ch2   O     Ch3   0.362          3     0.00
O      Ch2   Ch2   H     0.310          3     0.00
O      Ch2   Ch2   O     0.181          3   180.00
O      Ch2   Ch2   O     1.604          2     0.00

!! TFSI
Ft     Ct    St    Nt    0.702     3     0.00
Ft     Ct    St    Ot    0.038     3     180.00
St     Nt    St    Ct    0.389     1     0.00
St     Nt    St    Ct    1.805     2     0.00
St     Nt    St    Ct    1.658     3     0.00
St     Nt    St    Ot    1.241     3     180.00

!! FPFSI
Cf2    So2   N     Sof   1.581     1     180.00
Cf2    So2   N     Sof   0.170     2     0.00
Cf2    So2   N     Sof   0.246     3     180.00
Cf3    Cf2   So2   N     0.443     3     0.00
Cf3    Cf2   So2   Oso   0.647     3     0.00
```

```
F2     Cf2   So2   N     0.027      3     180.00
F2     Cf2   So2   Oso   0.313      3     180.00
F3     Cf3   Cf2   F2    0.112      3     180.00
F3     Cf3   Cf2   So2   0.080      3     0.00
Oso    So2   N     Sof   0.121      3     180.00
So2    N     Sof   F1    1.332      1     180.00
So2    N     Sof   F1    1.163      2     0.00
So2    N     Sof   F1    0.975      3     180.00
So2    N     Sof   F1    0.243      4     0.00
So2    N     Sof   Osf   0.281      3     0.00


!!!!!!!!!!!!!!!!!!!!!!!!!!!!!!!!!!!!!!!!!!!!!!!!!!!!!!!!!!!!!!!!!!!!
!!!!!!!!!!!!!
!********* V(Lennard-Jones) = Eps,i,j[(Rmin,i,j/ri,j)**12 -
2(Rmin,i,j/ri,j)**6]
!
!epsilon: kcal/mole, Eps,i,j = sqrt(eps,i * eps,j)
!Rmin/2: A, Rmin,i,j = Rmin/2,i + Rmin/2,j
!
!atom  ignored    epsilon      Rmin/2   ignored   eps,1-4
Rmin/2,1-4
!!!!!!!!!!!!!!!!!!!!!!!!!!!!!!!!!!!!!!!!!!!!!!!!!!!!!!!!!!!!!!!!!!!!
!!!!!!!!!!!!!

!! CATION
Li   0.0   -0.0005   1.6107   0.0   -0.00025   1.6107

!! G4
Ch2   0.0   -0.0660   1.9640   0.0   -0.0330    1.9640
O     0.0   -0.1400   1.6280   0.0   -0.0700    1.6280
H     0.0   -0.0300   1.4030   0.0   -0.0150    1.4030
Ht    0.0   -0.0300   1.4030   0.0   -0.0150    1.4030
Ch3   0.0   -0.0660   1.9640   0.0   -0.0330    1.9640

!! TFSI
Nt   0.0   -0.051   1.824   0.0   -0.0255   1.824
St   0.0   -0.075   2.291   0.0   -0.0375   2.291
Ot   0.0   -0.063   1.944   0.0   -0.0315   1.944
Ct   0.0   -0.020   1.768   0.0   -0.0100   1.768
Ft   0.0   -0.016   1.490   0.0   -0.0080   1.490

!! FPFSI
N    0.0   -0.051   1.824   0.0   -0.0255   1.824
So2  0.0   -0.075   2.291   0.0   -0.0375   2.291
Sof  0.0   -0.075   2.291   0.0   -0.0375   2.291
Oso  0.0   -0.063   1.944   0.0   -0.0315   1.944
Osf  0.0   -0.063   1.944   0.0   -0.0315   1.944
Cf2  0.0   -0.020   1.768   0.0   -0.0100   1.768
Cf3  0.0   -0.020   1.768   0.0   -0.0100   1.768
F1   0.0   -0.016   1.490   0.0   -0.0080   1.490
F2   0.0   -0.016   1.490   0.0   -0.0080   1.490
F3   0.0   -0.016   1.490   0.0   -0.0080   1.490

!! DRUDE Particles
```

```
DCh  0.0    0.0     0.0     0.0    0.0      0.0
DO   0.0    0.0     0.0     0.0    0.0      0.0
DOt  0.0    0.0     0.0     0.0    0.0      0.0
DSt  0.0    0.0     0.0     0.0    0.0      0.0
DNt  0.0    0.0     0.0     0.0    0.0      0.0
DCt  0.0    0.0     0.0     0.0    0.0      0.0
DFt  0.0    0.0     0.0     0.0    0.0      0.0
DCf  0.0    0.0     0.0     0.0    0.0      0.0
DOs  0.0    0.0     0.0     0.0    0.0      0.0
DSo  0.0    0.0     0.0     0.0    0.0      0.0
DN   0.0    0.0     0.0     0.0    0.0      0.0
DF1  0.0    0.0     0.0     0.0    0.0      0.0
DF2  0.0    0.0     0.0     0.0    0.0      0.0
DF3  0.0    0.0     0.0     0.0    0.0      0.0


NBFIX
Li Nt   -0.00505  3.8    -0.002525 3.8
Li N    -0.00505  3.8    -0.002525 3.8
Li Ft  -0.00283  3.4007 -0.001414 3.4007
Li F1  -0.00283  3.4007 -0.001414 3.4007
Li F2  -0.00283  3.4007 -0.001414 3.4007
Li F3  -0.00283  3.4007 -0.001414 3.4007

END
```

Atomic charges and polarizabilities

G4

| Atom | Charge [e] | Polarizability [Å$^3$] |
|---|---|---|
| Ch3 | -0.06000 | 1.547 |
| Ht | 0.06500 | 0.000 |
| O | -0.27000 | 0.791 |
| Ch2 | 0.00500 | 1.232 |
| H | 0.06500 | 0.000 |

Cation

| Atom | Charge [e] | Polarizability [Å$^3$] |
|---|---|---|
| Li | 1.00000 | 0.000 |

TFSI

| Atom | Charge [e] | Polarizability [Å$^3$] |
|---|---|---|
| Ot | -0.38930 | 1.360 |
| St | 0.59280 | 0.500 |
| Nt | -0.48540 | 1.450 |
| Ct | 0.13910 | 1.050 |
| Ft | -0.07020 | 0.600 |

FPFSI

| Atom | Charge [e] | Polarizability [Å$^3$] |
|---|---|---|
| Cf2 | 0.10644 | 1.050 |
| F2 | -0.11178 | 0.600 |
| So2 | 0.82217 | 0.500 |
| Oso | -0.45762 | 1.360 |
| N | -0.50677 | 1.450 |
| Sof | 0.87874 | 0.500 |
| Osf | -0.44608 | 1.360 |
| F1 | -0.24359 | 0.600 |
| Cf3 | 0.39205 | 1.050 |
| F3 | -0.13936 | 0.600 |
